# Co-delivery of doxorubicin and conferone by novel pH-responsive β-cyclodextrin grafted micelles triggers apoptosis of metastatic human breast cancer cells

**DOI:** 10.1038/s41598-021-00954-8

**Published:** 2021-11-02

**Authors:** Akram Rahmani, Fariborz Rahimi, Mehrdad Iranshahi, Houman Kahroba, Amir Zarebkohan, Mehdi Talebi, Roya Salehi, Hassan Zavvar Mousavi

**Affiliations:** 1 Department of Applied Chemistry, Faculty of Chemistry, Semnan University, Semnan, Iran; 2 Department of Electrical Engineering, University of Bonab, Bonab, Iran; 3 Faculty of Pharmacy, Mashhad University of Medical Sciences, Mashhad, Iran; 4 Molecular Medicine Research Center, Biomedicine Institute, Tabriz University of Medical Sciences, Tabriz, Iran; 5 Department of Molecular Medicine, Faculty of Advanced Medical Sciences, Tabriz University of Medical Sciences, Tabriz, Iran; 6 Department of Medical Nanotechnology, Faculty of Advanced Medical Sciences, Tabriz University of Medical Sciences, Tabriz, Iran; 7 Department of Applied Cell Science, Faculty of Advanced Medical Sciences, Tabriz University of Medical Sciences, Tabriz, Iran; 8 Drug Applied Research Center and Department of Medical Nanotechnology, Faculty of Advanced Medical Sciences, Tabriz University of Medical Sciences, Tabriz, Iran; 9 Department of Chemistry, Faculty of Science, University of Guilan, P.O. Box 41335-1914, Rasht, Iran

**Keywords:** Biochemistry, Chemical biology, Chemistry, Materials science

## Abstract

Adjuvant-aided combination chemotherapy is one of the most effective ways of cancer treatment by overcoming the multidrug resistance (MDR) and reducing the side-effects of anticancer drugs. In this study, Conferone (Conf) was used as an adjuvant in combination with Doxorubicin (Dox) for inducing apoptosis to MDA-MB-231 cells. Herein, the novel biodegradable amphiphilic β-cyclodextrin grafted poly maleate-co-PLGA was synthesized by thiol-ene addition and ring-opening process. Micelles obtained from the novel copolymer showed exceptional properties such as small size of around 34.5 nm, CMC of 0.1 μg/mL, and cell internalization of around 100% at 30 min. These novel engineered micelles were used for combination delivery of doxorubicin-conferone with high encapsulation efficiency of near 100% for both drugs. Our results show that combination delivery of Dox and Conf to MDA-MB-231 cells had synergistic effects (*CI* < 1). According to cell cycle and Annexin-V apoptosis analysis, Dox-Conf loaded micelle significantly induce tumor cell apoptosis (more than 98% of cells population showed apoptosis at *IC*_*50*_ = 0.259 μg/mL). RT-PCR and western-blot tests show that Dox-Conf loaded β*CD-g-PMA-co-PLGA* micelle induced apoptosis via intrinsic pathway. Therefore, the unique design of multi-functional pH-sensitive micelles open a new perspective for the development of nanomedicine for combination chemo-adjuvant therapy against malignant cancer.

## Introduction

Combination therapy has been used to improve the therapeutic outcomes and deal with the incidence of multi-drug resistance in cancer treatment. However, the high toxicity of multiple anticancer drugs to healthy tissues considers as a major concerns. As capable alternatives to chemo agents, non-chemo drugs like natural driven adjuvants to conventional anti-tumor therapeutics, proposing a harmless and economic approach for combination therapy. Adjuvants are compounds that reduce the side-effects of anticancer drugs a decrease in the required therapeutic dose while keeping its desired therapeutic effects or even increasing it^1^. Various adjuvants were utilized for Doxorubicin (Dox) combination therapy, such as orange peel and naringin extract^2^, curcumin^3^, quinacrine^4^ and conferone^5,6^. Conferone (Conf) is an extraction of various parts (such as root and fruit) of *Ferula* class herbs. This adjuvant has noticeable anticancer and anti-angiogenic properties. Furthermore, when used in combination with Dox, it is able to increase Dox-intracellular uptake and accumulation in cells by stopping Dox efflux via P-glycoprotein (P-gp) suppression. Also, previous studies showed that conferone had a synergic effect in combination with Dox. Conferone application is limited because of its hydrophobic nature, which leads to low bioavailability and cellular uptake^7^. The best approach to solve this problem seems to be a simultaneous delivery system of Dox and conferone to cancer cells.

The copolymeric micelles are suitable drug delivery systems that can solve bioavailability problems related to hydrophobic drugs. They are also capable of simultaneous hydrophobic and hydrophilic drug delivery. Also, the other principal advantages of copolymeric micelles are: minor size, passive targeting via the enhanced permeability and retention effect (EPR), extensive time of circulation in the body, stability (as the thermodynamically and kinetically), and opportunity of micelle surfaces manipulation^8,9^. The burst release of drugs from micelles and the insensitivity to cancer cells are the failures of micellar systems in cancer drug delivery^10^. Since the micro-environment of cancer cells has acidic pH^11,12^, pH-sensitive nano-micelles can solve both these problems and be one of the favorite elections for combination targeted chemotherapy.

Beta-cyclodextrin (βCD) is a cyclic oligomer of glucose with a cage-like structure, and lipophilic inner cavities and hydrophilic outer surfaces^13^. βCD has fascinated researchers in the field of drug delivery systems design, due to a collection of properties including: biocompatibility, biodegradability, capability of inclusion-complex formation, large and nonpolar cavity space that traps drugs, and improved drug water solubility^14–16^. Moreover, βCD has seven primary hydroxyl groups that could react with different polymers to form a star copolymer. Star-shaped copolymers have lesser viscosity, smaller critical micelle concentration (CMC), lower hydrodynamic radius, and higher stability which leads to lower coefficients of diffusion^17–19^. Therefore, βCD was added to our copolymer structure by grafting βCD to the poly maleate section (as the pH-sensitive shell of copolymeric micelles), in order to reach a lower CMC value and higher drug loading capacity (both of hydrophobic and hydrophilic drugs). This βCD-grafted polymer or star-like shaped copolymer is joined to PLGA as the core (hydrophobic part) of micelles, for increased stability and biodegradability of the copolymer. **Hypothesis:** Consequently, it is hypothesized that the novel pH-sensitive βCD-grafted PLGA-based copolymer will create micelles with very low CMC and size, because of the special multifunctional amphiphilic structure design. As a result of its multifunctional structure, it is expected to see an enhancement of multi-drug loading capacity, conferone solubility and bioavailability in aqueous environments. It is also anticipated that this novel engineered micelle shows a pH-responsive sustained release of drugs. Most importantly, we hypothesize that Dox-conferone in this new nano-combination form will show a synergic effect and induce apoptosis in the cancer cells.

## Materials and methods

### Materials

Beta cyclodextrin, maleic anhydride, glycolide, lactide, tin (II) octoate, azo-bis-isobutyronitrile (AIBN), polyvinyl alcohol (89,000–98,000 Da), Tween 20 and propidium iodide, were purchased from sigma-Aldrich (USA). 2-mercapto ethanol, Sodium hydride (60%, suspension in paraffin) and all the solvents (Toluene, dimethyl sulfoxide, N,N-dimethylformamide, acetone, diethyl ether), were purchased from Merck, Germany. MTT dye [3-(4,5-dimethyl thiazol-2-yl)-2,5-diphenyl tetrazolium bromide] was obtained from Alfa Aesar, Thermo fisher scientific, Heysham, UK. Penicillin–Streptomycin (Pen-Strep, 100x) was bought from Serana Europe GmbH, Germany. MDA-MB-231 human breast cancer cell line was purchased from Pasteur institute. Fetal bovine serum, Trypsin–EDTA 0.25% (1X) and Roswell Park Memorial Institute 1640 growth medium (RPMI 1640) were provided from Gibco, Life Technologies limited, UK. Doxorubicin hydrocholoride (Ebedoxo) was purchased from EBEWE pharma, Austria. Conferone was provided by Iranshahi et al.^20^ that was extracted from the roots of *Ferula flabelliloba*. Ribonuclease A, was bought from Thermoscientific, EU, Lithuania. The Apoptosis kit of ApoFlowEx FITC Kit) was bought from EXBIO Praha, a.s., Czech Republic. TRIzol was purchased from Life Technologies, USA. SYBR Green Master Mix, RealQ Plus, 2 × Master Mix Green, was obtained from Ampliqon, Denmark; and primers were obtained from Eurofin, Germany.

### Instruments

To determine chemical structure of the synthetic copolymer, a Fourier transform infrared spectrometer, FTIR, Bruker, Tensor 27, Germany, in the range of 400–4000 cm^−1^ (the copolymer as KBr tablets) and ^1^HNMR and ^13^CNMR spectrometer by Bruker, spectra spin 400 MHz, Leipzig, Germany (DMSO-d_6_ as solvent) were used. For elemental analysis (C, H, N and S %) of copolymer, a combustion CHNS analyzer, HromLab Costech elemental analyzer, ECS 4010, Germany, was used. A differential scanning calorimeter (DSC), NETZSCH DSC 200 F3 Maia, Germany, with pure nitrogen purging gas and closed pan aluminum crucible, was used for DSC analysis and determination of glass transition temperature (***T***_***g***_) of the copolymer. In this test, 9 mg of copolymer was heated to above its melting point, in order to remove its thermal history. Subsequently, the sample was cooled to − 90 °C using liquid nitrogen (rate: 10 °C/min). Then, the sample was heated until 250 °C, in the second run. For preparing of copolymeric micelles an ultrasonic probe, SYCLON, SKL-500 II DN, Ningbo Haishu Sklon electronic instrument Co., Ltd. (China), was used. An Amicon centrifugal filter, Ultra-15, MWCO: 50 KDa, Millipore, Darmstadt, Germany, was used for micelle solution centrifuging.

A field emission scanning electron microscope, MIRA3-XMU TESCAN FESEM (Czech) was utilized for determining morphology and size of copolymeric micelles. The mean diameter of about 150 micelles in SEM image, was calculated by image analysis software, Image-Pro plus 4.5; Media Cybernetics, Silver Spring, MD. A dynamic light scattering (DLS) device, DLS-Zetasizer Nano ZS90, Malvern Instruments, Malvern (UK), was used for determining size and zeta potential of micelles. A spectrofluorometer system, Jasco FP-750 (japan), was used for critical micelle concentration study. Drug amounts in loading and release study, were measured with UV–visible spectrophotometer, UV 160-Shimadzo, Japan. In order to prepare image of cells in intracellular uptake test, a fluorescence microscope, Nikon E1000M, Tokyo (Japan) armed with a Planapo apochromatic objectives, Nikon, Tokyo (Japan) was used. For measuring intracellular uptake, cell cycle, and apoptosis tests, a FACS calibur flow cytometer, Becton Dickinson Immuno-cytometry Systems, San Jose, CA (USA) was utilized. For quantifying of total RNA, a NanoDrop system, ND-1000 (Australia), was used. For cDNA synthesis, a PeQlab (UK) device was employed; and for real-time PCR process, a Roche, Light Cycler 96 (USA) was used. A spectrophotometer, Bibby Scientific Ltd, Beacon Rd (UK) was used for protein measurement in western blotting. Finally, an Amersham Imager 600 system, GE Healthcare Life Sciences, Eindhoven (the Netherlands), was utilized for measurements of protein bands in western blot test.

### Block-Copolymer Synthesis

#### Hydroxy terminated poly maleic anhydride synthesis

The synthesis method of poly maleic anhydride with hydroxy termination (*PMA-OH*), was reported in our previously published paper^6^. Briefly, after dissolving of 3.93 mg maleic anhydride (*MA*) in 60 mL toluene under refluxing and nitrogen purging, 3.5 mL 2-mercapto ethanol (*ME*) was poured into the solution by a syringe. After temperature reached 110 °C, 0.147 g of Azobis isobutyronitrile (*AIBN*) in dry toluene, was added to the flask via injection. Twenty hours was allowed for the completion of the reaction. The light-yellow product was then purified and precipitated by solvent/antisolvent system (respectively acetone/toluene). The prepared *PMA-OH* was then dried by freeze-dryer.

#### Preparation of beta cyclodextrin grafted *PMA-OH*

In order to activate beta-cyclodextrin (βCD), 0.98 g (equivalent to 0.00086 mol) of βCD was dissolved in 40 mL of dry dimethyl formamide (dry DMF) in a two-necked flask under a nitrogen atmosphere and stirring. After the complete dissolution of βCD, 0.17 g (equivalent to 0.007 mol) of NaH in the solid state, was added to the reaction solution. After completing of βCD activation at room temperature (24 h), the reaction flask was placed in an oil bath and the temperature was raised to 100 °C. Then, 0.58 g of *PMA-OH* (equivalent to approximately 0.003 mol MA) solution in dry DMF was added to the contents of the flask, dropwise, under the nitrogen purging and stirring. The reaction was continued for 24 h at 100 °C, under nitrogen atmosphere and stirring. After 24 h, the reaction mixture was poured into 150 mL of a mixture of acetone, acetic acid and water (100 mL acetone, 10 mL acetic acid and 50 mL distilled water) and stirred for 30 min to inactivate and wash the excess or unreacted NaH. Then, the product was precipitated again with pure acetone. The product of the second stage (βCD grafted hydroxy terminated poly maleate = β*CD-g-PMA-OH*), which was a creamy pale-yellow precipitate, was dried and stored.

#### Preparation of β*CD-g-PMA-co-PLGA*

β*CD-g-PMA-OH* (0.4 g, approximately equal to 0.0003 mol), lactide (1.5 g, 0.01 mol), and glycolide (0.5 g, 0.0043 mol) were poured into a two-necked flask. After complete melting of the material at 120 °C, under the nitrogen atmosphere and stirring, a certain amount of tin (II) octoate, Sn (Oct)_2_, (1–3% w/w of the total monomers) as the catalyst, was added to the contents of the flask. The mixture was stirred at 120 °C for 24 h. The prepared final copolymer (β*CD-g-PMA-co-PLGA*) was then purified by solvent/antisolvent precipitation (Dichloromethane/Diethyl ether) and dried by freeze-dryer.

FTIR, ^1^HNMR, ^13^CNMR, CHNS and DSC analyses were used for investigating chemical structure and physicochemical properties of copolymer.

### Degradation test of copolymer

For investigating in-vitro biodegradability of the copolymer, it was examined at two different pH environments. For each experiment, 5 mg of copolymer was dispensed in 2 mL of PBS at pH values of 7.4 and 5.5 and incubated at 37 °C in a shaker-incubator. For each of the pH values, and each specified time interval, two repetition were considered. In other words, after each specified time intervals (7, 11, 16, 21 and 30 day), four samples were centrifuged (12,000 rpm, 30 min) and the supernatants were separated from the copolymer precipitants. The supernatants pH, were measured, separately. After complete drying of copolymer precipitants, they were weighed and then analyzed by FTIR. The supernatants pH variation and weight loss percentage (***WL %***) of copolymer in different time intervals were calculated using Eq. (1)^21^.

1 $$WL \left(\%\right)=\frac{{W}_{i}-{W}_{t}}{{W}_{i}}\times 100$$

where *W*_*i*_ is the initial sample weight and *W*_*t*_ is the sample weight at time t.

### Determination of critical micelle concentration (CMC)

Spectro fluorometry method with pyrene probe was used to find critical micelle concentration (CMC) of the copolymer. One μL of pyrene solution (1 mg of pyrene in 10 mL of acetone) was added into dark flasks. After evaporation of acetone, the copolymer solution in dimethyl sulfoxide, DMSO, was poured into flasks. The final volume of flask was reached to 20 mL (5 mL copolymer solution and 15 mL deionized water) and copolymer final concentration was adjusted at 0.05, 0.1, 0.5, 1, 2.5, 5, 10, 25, 50, 100, 250, 500, 1000 μg/mL. The flasks were micellized by ultrasound probe and then incubated in a shaker incubator at 37 °C for 18 h, in order to balance pyrene partition between two phases. After cooling the samples to room temperature, the emission spectra of pyrene in each of the samples was studied by a spectrofluorometer. The excitation and emission wavelengths for pyrene spectra were 334 nm and 373 nm (*I*_*1*_) and 393 nm (*I*_*3*_), respectively.

### Preparation of blank and drug-loaded micelles

Blank β*CD-g-PMA-co-PLGA* micelles were prepared by adding of copolymer solution (200 mg of copolymer in 6 mL of DMSO) dropwise into polyvinyl alcohol (PVA) solution (20 mL, 1% w/v), under sonication in an ice bath. Then the blank β*CD-g-PMA-co-PLGA* micelle solution was centrifuged (4500 rpm, 10 min) by Amicon centrifugal filter (MWCO: 50 KDa). In order to remove the residue of DMSO, the blank β*CD-g-PMA-co-PLGA* micelles suspension in 2 mL deionized water was transferred into a dialysis membrane (CelluSep H1, MWCO: 2000 Da) and purified by dialysis method against the deionized water as external phase for 24 h. The old external solution was removed several times and replaced with fresh deionized water. The purified blank micelles inside of the dialysis membrane was freeze-dried and kept at − 24 °C.

In order to prepare Doxorubicin (**Dox**) loaded β*CD-g-PMA-co-PLGA* micelles, the copolymer solution (200 mg copolymer in 6 mL DMSO) was added dropwise to PVA solution (20 mL, 1% w/v) containing 20 mg **Dox**, and then was sonicated by ultrasound probe, while the pH of micelle solution was adjusted at 7.4 by sodium hydroxide (NaOH) solution. After centrifuging by Amicon centrifugal filter (4500 rpm, 10 min), the **Dox** loaded β*CD-g-PMA-co-PLGA* micelles were collected, dried and stored at − 24 °C. After centrifuging, the supernatant was utilized to determine **Dox** loading percentage.

For loading conferone (**Conf**) in β*CD-g-PMA-co-PLGA* micelles, first, 200 mg of copolymer and 20 mg of conferone were dissolved in 6 mL of DMSO. Then, the prepared solution was added to PVA solution similar to **Dox** loaded micelle preparation. After ultrasonication and centrifuging micelles, the **Conf** loaded β*CD-g-PMA-co-PLGA* micelles were dried and stored at − 24 °C. The supernatant solution was used to quantify drug loading percentage.

The co-drug loaded β*CD-g-PMA-co-PLGA* micelles, was prepared by gradually adding of copolymer and **Conf** (200 mg and 10 mg, respectively) solution in DMSO (6 mL), to PVA solution containing **Dox** (10 mg of **Dox**/20 mL PVA), with sonication in dark and ice bath. Acidity of solution was adjusted at 7.4. The following steps were done similar to **Dox** loading process.

Drug loading and release amounts were determined by UV–Vis spectrophotometer, for which λ_max_ of **Dox** and **Conf** was 480 and 324 nm, respectively. Then, the drug encapsulation efficiency (*DEE %*) was obtained using Eq. (2)^22^:

2 $$\mathrm{DEE }\left(\mathrm{\%}\right)= \frac{\mathrm{Mass\,of\,drug\,in\,nanocarrier}}{\mathrm{Initial\,mass\,of\,feed\,drug}}\times 100$$

### Characterization of copolymeric micelles

The size, morphology and zeta potential of blank micelles were investigated by SEM and DLS-Zeta analyses. Moreover, FTIR spectra and zeta potential of the blank and co-drug loaded micelles were studied in order to confirm the drug loading into micelles.

### In-Vitro study of drug release

First, 1 mg of dried single- and co-drug loaded β*CD-g-PMA-co-PLGA* micelles were weighted in microtubes and then were suspended in 2 mL of sink solution with two pH values, separately. Due to lower solubility of conferone in PBS buffer solutions, the release study was conducted in sink solution contained 0.5% DMSO, 0.5% Tween 20 and 99% PBS (two pH values of 5.5 and 7.4) to improve conferone solubility. Then, the microtubes (contain the samples) were placed in a shaker-incubator at 37 °C. After different time intervals (1, 2, 3, 7, 9, 11, 14 and 16 day), the microtubes containing samples were centrifuged (12,000 rpm, 25 min). After supernatant collection in each time interval, 2 mL of fresh sink solution was added to precipitant and sample was re-incubated in a shaker-incubator at 37 °C. The drugs amount in collected supernatant were detected by UV–Vis spectrophotometer and then release percentage of drugs were measured using Eq. (3)^23^. All stages of this test were duplicated for each pH value.

3 $$Drug\,release \left(\%\right)= \frac{{\sum }_{0}^{t}(amount\,of\,drug\,in\,release\,medium\,at \,time\,t)}{amount\,of\,drug\,loaded\,in \,nanocarrier }\times 100$$

### Nano-formulations cytotoxicity study by MTT method

The cytotoxicity of all formulations, **Dox** loaded β*CD-g-PMA-co-PLGA* micelles (**BD**), **Conf** loaded β*CD-g-PMA-co-PLGA* micelles (**BC**), **Dox** and **Conf** loaded or co-drug loaded β*CD-g-PMA-co-PLGA* micelles (**B2D**), free Doxorubicin (**Dox**), free conferone (**Conf**), free Doxorubicin and conferone combination (**2D**) and blank β*CD-g-PMA-co-PLGA* micelles (**PB**), on MDA-MB-231 cells, were examined by MTT analysis. The cells were cultured in 96-well plates (7000 cell per well), that each well contained 200 μL of RPMI medium with 10% FBS. The cultured cells were incubated at 37 °C with 5% CO_2_. After 48 h, the cells were treated with all the formulations with several concentrations and incubated again. The treatment was done triplicate. A series of un-treated cells were selected as the control group. After 48 h, the cells were washed with PBS. Then, 150 μL complete RPMI medium with MTT solution (50 μL of 2 mg MTT in 1 mL of PBS) were added per well, in dark condition. The palates were incubated again for 4 h. Subsequently, the medium of each wells was replaced with 200 μL of DMSO. After complete dissolving of formazan crystals in DMSO, the plates were situated in a microplate ELISA reader to measure the absorbance of wells content, at 492/630 nm.

After calculation of cell viability (Microsoft *Excel*) the *IC*_*50*_ dosage of all formulations were estimated using *Graph pad prism* software. Then, the combination index (*CI*) of co-drug forms, were obtained by *CompuSyn* V.1 software. In *CI* analysis, *CI* < 1 shows synergism, *CI* = 1 and *CI* > 1 displays additive effect and antagonism, respectively.

### Study of nano-formulations intracellular uptake

In order to preparation of rhodamine-B-labeled blank β*CD-g-PMA-co-PLGA* micelles, first, 10 mg of copolymer and 0.1 mg of rhodamine-B (**RB**) was dissolved in 1 mL of DMSO. Then the prepared solution was added dropwise to 4 mL of PVA solution (1% w/v), under sonication in an ice bath and dark condition. After centrifuging (8000 rpm, 15 min) of micelle solution, the supernatant was removed. The precipitated rhodamine B-labeled blank β*CD-g-PMA-co-PLGA* micelles were washed by distilled water and centrifuged several times for complete removal of unloaded rhodamine-B. The precipitated rhodamine B-labeled blank β*CD-g-PMA-co-PLGA* micelles were dispersed in deionized water (1 mL) and were kept at − 24 °C. For preparing of co-drug loaded β*CD-g-PMA-co-PLGA* micelles labeled by rhodamine-B, 10 mg of copolymer, 0.5 mg of **Conf** and 0.1 mg of **RB**, were dissolved in 1 mL DMSO and added to PVA 1% w/v solution containing 0.5 mg of **Dox**, under sonication. Then polymer/conf solution containing **RB** was added dropwise to PVA/Dox solution under sonication in an ice bath and dark condition. Next steps were done like rhodamine B-labeled blank β*CD-g-PMA-co-PLGA* micelles preparation procedure.

Subsequently, the MDA-MB-231 cells were cultured in the 6-well palates (with population of 2 × 10^5^ cell per well) in complete RPMI medium containing 10% FBS. After incubation at 37 °C with 5% CO_2_ for 48 h, the cells were treated with rhodamine B-labeled blank and co-drug loaded β*CD-g-PMA-co-PLGA* micelles for 0.5, 1.5 and 3 h. The un-treated cells were chosen as the control group. Next, the cells were washed with PBS and trypsinized. After centrifuging (1500 rpm, 5 min) the cells were washed with PBS again. In order to quantify the fluorescent intensity of internalized rhodamine-B-labeled blank and co-drug-loaded β*CD-g-PMA-co-PLGA* micelles, the washed cells were dispersed in PBS (about 300 μL) and analyzed with FACS Calibur flow cytometer. For qualitative analysis, fluorescent imaging by a fluorescence microscope was also utilized. The images of treated cells (with rhodamine B-labeled co-drug-loaded β*CD-g-PMA-co-PLGA* micelles), were prepared similar to our previously published paper^6^.

### Study of nano-formulations effect on cell cycle

The MDA-MB-231 cells (3 × 10^5^ cell per well) were seeded in 6-well plates and incubated (48 h). Then, all formulations with *IC*_*50*_ dosage, were applied for treatment of the cells. The un-treated cells were selected as the control group. Then, the plates were incubated for another 48 h. After moving the medium of each treated cells into separate tubes, the cells were washed with PBS, trypsinized and transferred back to corresponding tubes. As soon as centrifuging of tubes and removing of their supernatant were completed, the cells were dispersed in PBS (700 µL) and centrifuged again. Then, the supernatants were discarded and the cells were dispersed in 300 µL cold PBS. For fixing of cells, 700 µL of cold ethanol 70%, was poured to each of the tubes and mixed. The tubes were located at 4 °C, in dark condition for 3 days. Then, the samples were centrifuged and after removing of supernatants, the cells were dispersed in 300 µL of PBS. Next, after adding 10 µL of Ribonuclease-A (10 mg/mL) and 45 min incubating, 10 µL propidium iodide (1 mg/mL) was added to each of the samples and vortexed. After 10 min incubation at the room temperature and dark condition, the cells were examined by FACS Calibur flow cytometer for estimation of cell cycle phases.

### Apoptosis study induced by nano-formulations

The effect of formulations on MDA-MB-231 cells were studied by Exbio apoptosis kit of Annexin V-FITC/PI. As soon as reaching 60% confluency, the cells were cultured (1 × 10^5^ cell per well) in 6-well plates and were incubated (48 h). The nano-formulations (**PB**, **B2D**, **BD** and **BC**) with *IC*_*50*_ doses were used for treatment of the cells. After 48 h, the medium of wells were transferred to separate tubes. Then, the cells were washed with PBS, collected, and transferred back into the corresponding tubes. The tubes were centrifuged and the cells were washed with PBS two times, after removing of supernatants. After washing by annexin binding buffer (BB), the cells were dispersed in of binding buffer (100 μL). Then, Annexin V-FITC (5 μL) and of propidium Iodide (PI, 5 μL), were added to cell dispersions and vortexed gently. After incubation at room temperature in dark condition (15 min), the samples were centrifuged and the supernatants were discarded. Finally, the cells were suspended in binding buffer (100 μL) and were evaluated by a FACS Calibur flow cytometer. The un-treated unstained cells were selected as the auto-fluorescence control group.

### Real-time PCR analysis

The MDA-MB-231 cells were seeded and treated with all formulations, like the protocol of the previous section. After 48 h, the cells were washed with PBS twice and then trypsinized and centrifuged. The supernatants were removed and the cells were dispersed in PBS (250 μL). According to TRIzol method for RNA isolation, RiboEx (750 μL) and then chloroform (200 μL) were poured into samples to lysis of cells and extract RNA. Following a short incubation (2 min, at room temperature), the samples were centrifuged (12,000 g, 20 min, 4 °C) and the upper aqueous layer (RNA phase), were separated. Then, isopropanol (500 μL) was added to separate RNA solution and the samples were centrifuged (12,000 g, 20 min, 4 °C) for precipitation of RNA. The precipitant was washed with ethanol (75%). After dissolving of the precipitant in DEPC-treated water, the RNA content of solution was determined by NanoDrop. In the next step, synthesis of cDNA (complementary DNA) was performed using Revert Aid Reverse Transcriptase Kit.

Finally, in order to perform quantitative PCR (qPCR) and investigate apoptotic pathway of treated cells, the samples were prepared as a mixture of SYBR Green Master Mix (5 μL, 2x), cDNA (2 μL), primer pair mix (5 pmol/μL) and deionized water (3 μL). This mixture was prepared for each of the formulations separately. In the PCR program, initial denaturation of samples was done for 15 min at 95 °C. Then, the run was proceeded at 95 °C for 15 s, which was repeated for 45 cycles. The annealing/extension stage was completed for 50 s at 60 °C. The sequences of used primers are presented in Table S1. The GAPDH was considered as the references gene. Lastly, the fold changes of genes expression were calculated by − ∆∆C_t_ method.

### Study of protein expression by western blot method

Like the previous section, the cells were cultured in 6-well plates and treated by co-drug loaded β*CD-g-PMA-co-PLGA* micelles (**B2D**, with *IC*_*50*_ dosage) and un-treated cells were considered as the control group. After 48 h incubation, the cells were washed with PBS and harvested. Then, radioimmunoprecipitation assay buffer (RIPA buffer) at 4 °C, was used for cell lysing. The RIPA buffer was composed of protease inhibitor cocktail (1 tablet), Tris–HCL (pH = 8, 500 µL), NaCl (0.08 g), EDTA (0.003 g), Sodium deoxycholate (0.025 g), Triton NP40 (10 µl, 1%) and SDS (0.01 g). Subsequently, the samples were centrifuged (12,000 rpm, 10 min, 4 °C) and the protein content of supernatant, was determined by a spectrophotometer, according to protocols of Bradford assay (Bio-Rad Laboratories, USA). The target fragments of proteins that were separated from the SDS-PAGE gel electrophoresis, were moved to the PVDF membrane (polyvinylidene difluoride membrane) and were blocked with 5% w/v of skim milk and 0.1% v/v of Tween 20 in tris buffered saline (TBS) for masking of unspecific bands. Specific primary antibodies were added to the blocked PVDF membranes that contained the target proteins and were incubated (overnight at 4 °C). After washing with TBS-T, the membranes were incubated with secondary antibodies, for 1 h at room temperature. The bands related to the target proteins were visualized using enhanced chemiluminescence detection kit (Thermo Fisher Scientific, the Netherlands) and were measured with Amersham Imager. Lastly, after normalizing of the outcomes of western blot using GAPDH expression as the control, the blots were calculated using Image J software, version 1.52n. The used primary and secondary antibodies were presented in our previously published paper^6^.

### Statistical analyses

The duplicate or triplicated outcomes of analyses, were presented as ± standard deviation (± SD) using Graph pad prism software, version-8 or Microsoft Excel (2019). The student’s t-test and ANOVA were used as statistical analyses for two-way and multiples comparisons, respectively. The statistically significant results had the *P* value lesser or equal to 0.05.

## Results and discussion

**Figure 1:**
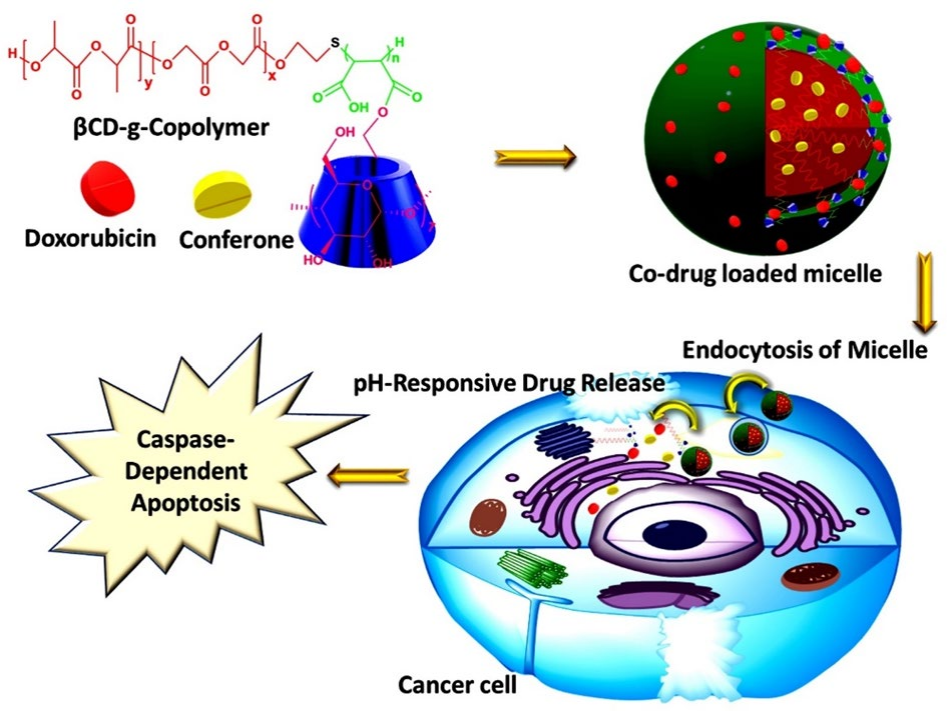
The graphical schematic of the general procedure.

The general procedure is briefly presented as a schematic illustration in Fig. 1.

### Designing and synthesizing of copolymer

**Figure 2:**
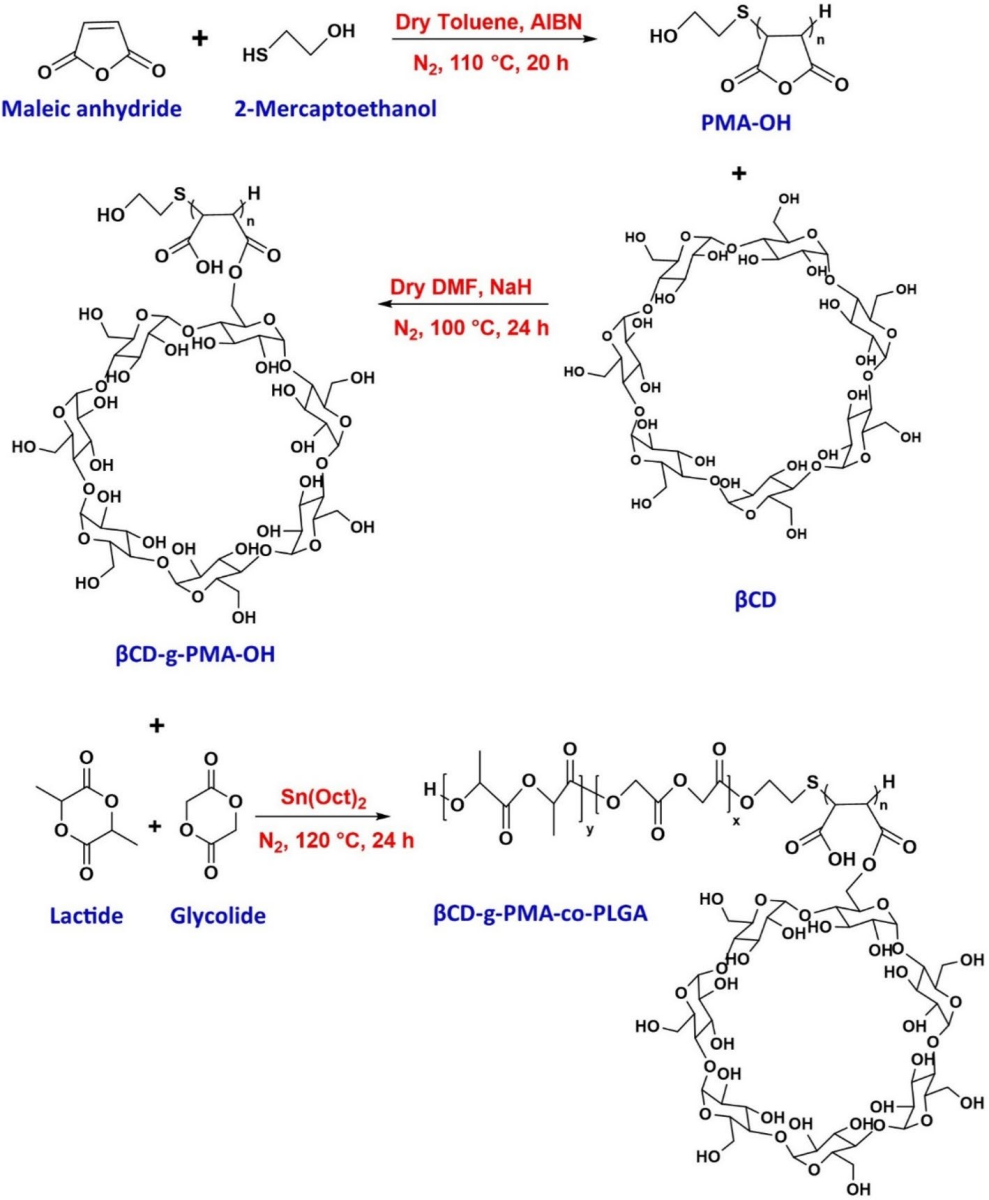
The synthesis pathway of copolymer in three stages: (top row) PMA-OH synthesis with radical thiol-ene addition and maleic anhydride (MA) polymerization, (middle row) βCD-g-PMA-OH synthesis by βCD-grafting with MA ring opening, and (bottom row) βCD-g-PMA-co-PLGA preparation with ring opening of lactide and glycolide.

As demonstrated in Fig. 2, the synthesis of pH-responsive βCD-g-poly maleate-co-PLGA was done in three stages. In the first stage, 2-mercaptoethanol (*ME*) and Maleic anhydride (*MA*) were polymerized with radical thiol-ene addition in the presence of AIBN as the initiator. The product of this stage is the hydroxy terminated poly maleic anhydride (*PMA-OH*). As a result of forming of ME radicals with initiator, the **C**=**C** band of MA was reacted radically with ^**•**^**S** end of thiol radical and then polymerized without any ring opening. The end **–OH** group of *PMA* was required for the last stage of synthesis.

In the second stage, the rings of PMA were opened with activated βCD (which was transferred to epoxy form by NaH) and then were esterificated. Esterification may be accomplished by one or more locations of hydroxyl groups of βCD and anhydride rings of PMA. Therefore, the β*CD-g-PMA-OH* was formed with one or more branches of PMA per one molecule of βCD. This βCD grafted polymer has a carboxylic acid in every unit of polymer that was required for pH-sensitivity of delivery system, formation of hydrophilic section of copolymer as the shell of micelles, and enhancing of water solubility of copolymer. About 1 g of product was obtained from this step (efficiency 64.1%).

In the final stage, the **–OH** end group of β*CD-g-PMA-OH*, with the catalyzing effect of Sn(Oct)_2_, caused ring openings of lactide and glycolide and their esterification to PLGA form (as the tail of copolymer and core of micelles). About 1.4 g of β*CD-g-PMA-co-PLGA* was obtained from this step (efficiency 58.3%).

### Characterization of copolymer

#### FTIR results

The FTIR spectra of βCD and all stages of synthesis are presented in Fig. S1 in the supplementary file and is enlarged for better visualization of details in Fig. S2.

The detailed explanation of FTIR spectra of *PMA-OH*, β*CD-g-PMA-OH* and β*CD-g-PMA-co-PLGA* were presented in supplementary file.

#### NMR results

**Figure 3:**
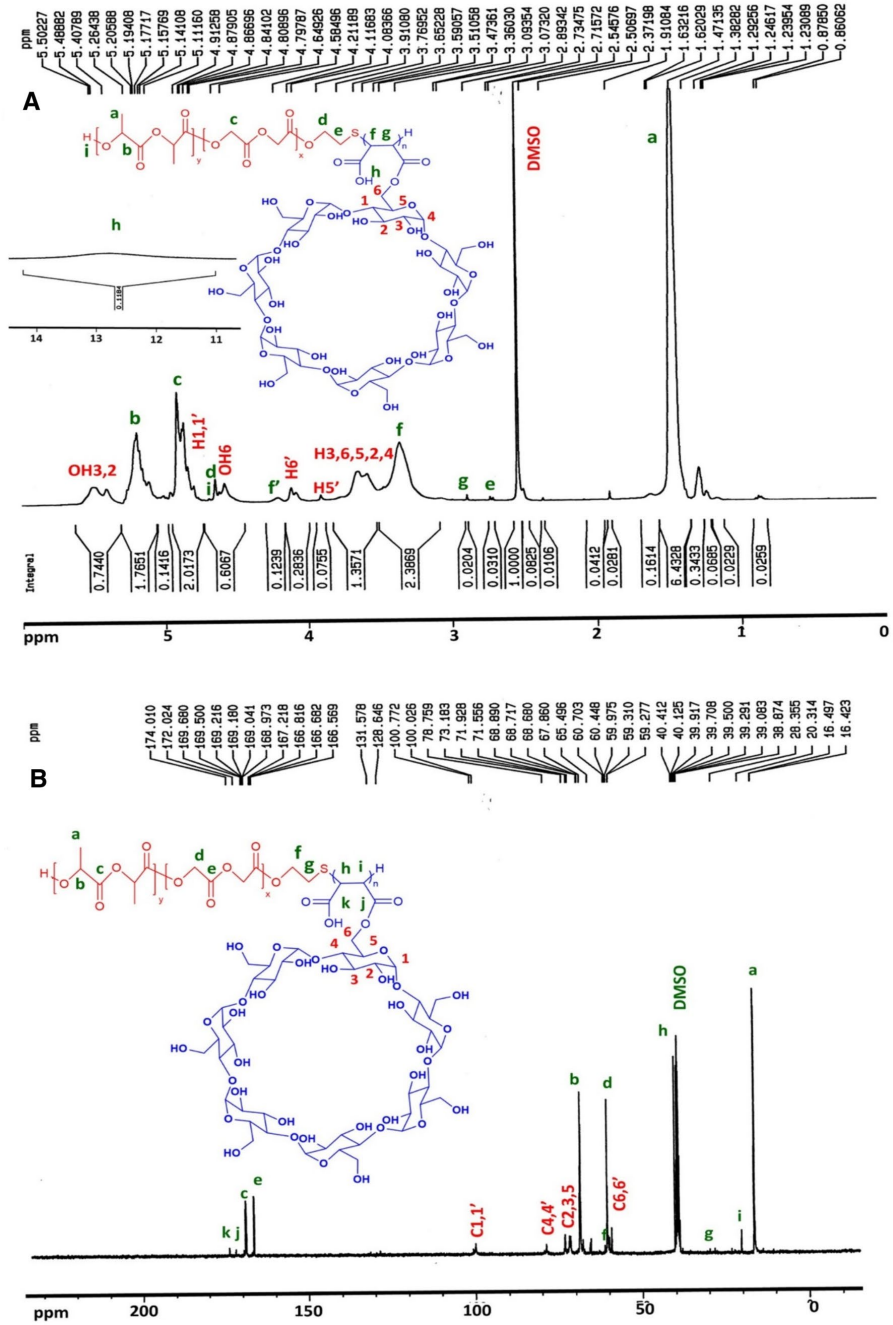
NMR Spectra of βCD-g-PMA-co-PLGA copolymer in DMSO-d_6_, (**A**) ^1^HNMR and (**B**) ^13^CNMR spectrum.

Results and detailed discussion of ^1^HNMR and ^13^CNMR spectra of *PMA-OH*, were presented in our previously published paper in detail^6^. The ^1^HNMR and ^13^CNMR spectra of β*CD-g-PMA-OH*, are shown in Fig. S3-A and S3-B, and the ^1^HNMR and ^13^CNMR spectra of β*CD-g-PMA-co-PLGA*, are presented in Fig. 3-A and 3-B, respectively. The detailed explanation of ^1^HNMR and ^13^CNMR spectra of β*CD-g-PMA-OH* and β*CD-g-PMA-co-PLGA* were presented in supplementary file.

The molecular weight of β*CD-g-PMA-co-PLGA* could not be investigated with gel permeation chromatography (GPC), due to the insufficient solubility of the copolymer in DMF solvent. Furthermore, because of the vigorous interaction of hydroxyls of the βCD with the GPC column, other researchers have also reported problems with calculating the molecular weight of their polymer by GPC^13,24^. Therefore, Eqs. (4) and (5) were used for determining molar mass (M_n_) of β*CD-g-PMA-co-PLGA*, with the aid of integrating of the peaks in ^1^HNMR spectrum^25^.

4 $${n}_{polymer}=\frac{{\sum }_{i=1 }^{m}\frac{{I}_{i}}{ {p}_{i}}}{m}$$

5 $${M}_{n}=n. (monomers\,molecular\,mass)$$

In the Eq. (4), "*m*" is the number of used signs of copolymer, and "*p*_*i*_" and "*I*_*i*_" are the number and integration of protons that pertained to *i*th peak of copolymer. The calculation of molar mass of copolymer are presented in supplementary file. The results of related calculations are reported in Table 1.

**Table 1:** M_n_ value for copolymer calculated using ^1^HNMR, with theoretical and spectrum-based calculated (by ^1^HNMR) molar ratio of βCD-g-PMA-co-PLGA copolymer sections.

M_n_ (g/mol)	LA: GL (mole %)		LA: GL: βCD-g-PMA (mole %)	
	Theoretical	Calculated (by ^1^HNMR)	Theoretical	Calculated (by ^1^HNMR)
4420.23	69.93: 30.07	68.18: 31.82	68.49: 29.45: 2.06	57.69:26.92: 15.39

#### Results of CHNS elemental analysis

For elemental analysis of copolymer by CHNS analyzer, 4.568 mg of copolymer was used. Results of test are presented in Table S2. According to data, the presence of sulfur (4.44% w), confirmed the existence of **–S–** linkage in copolymer structure. In the same way, the negligible amount of "**N**" (0.85% w) is probably related to impurities such as solvents residues (such as DMF). The CHNS time versus voltage plot are shown in Fig. S4.

#### Results of DSC study

Figure S5, presents the DSC or temperature against heat flow plot of copolymer. The ***T***_***g***_ or glass transition temperature of copolymer was determined with an endothermic peak in DSC plot, about 38.69 °C. Absence of ***T***_***m***_ or melting point of copolymer in the plot was a sign of amorphous structure without any crystallinity. This result is confirmed with DSC results of other studies related to PLGA-based polymers and copolymers. According to these reports, ***T***_***m***_ was not observed because of amorphous structure of PLGA, and also ***T***_***g***_ was reported to be between 35–65 °C, related to LA: GL ratio in PLGA structure (50–10% of LA in PLGA) that showed the ***T***_***g***_ was decreased with increasing of GL content of PLGA^26^.

#### Results of in-vitro degradation test of copolymer

Biodegradable copolymers encounter gradual degradation in contact of aqueous solution. In most polyesters, such as PLGA-based copolymers, hydrolysis of esteric-band and cleavage of copolymer is the main reason of degradation^27^. The produced soluble cleaved-copolymers and monomers such as lactic and glycolic acid are produced, that decreases pH of the solution. Therefore, timewise investigation of structure and weight of residual copolymer and pH of solution (that was in contact with copolymer during degradation test) are the suitable ways to determine degradation time and process. Results of degradation test are shown in Figs. S6-A, S6-B, S7 and S8. Fragmentation of the copolymer by degradation cause a gradual decrease in copolymer weight. Diagram of weight loss (*WL %*) of copolymer versus time is shown in Fig. S6-A. As shown in Fig. S6-A, copolymer initial weight decreased with time gradually. However, percentage of weight loss at pH = 7.4 was more than at pH = 5.5, that is probably due to the more hydrolysis of ester and carboxylic acid groups and subsequent more dissolution in PBS (with pH = 7.4). After 30 days, the *WL %* was reached to 19 and 20%, at pH = 7.4 and 5.5, respectively. As could be seen at day 30, the *WL* percentage at pH 5.5 excelled over the pH = 7.4, due to the initiation of major degradation process of copolymer (due to cleavage) rather than slight degradation (due to dissolution).

Variation of the pH of degradation medium are plotted versus time in Fig. S6-B. Hydrolysis of carboxylic acid groups related to maleate block, caused the initial sharp decrease in pH. After 7 days, pH-decrease slowed down, as a result of slight copolymer degradation. Finally, after 30 days, pH value reached to 6.2 and 3.8 for initial pH = 7.4 and 5.5, respectively. These pH values are similar to other reports about PLGA or PLGA-based copolymers that were about pH ≈ 5.48–7.4 with initial neutral pH^28,29^. However, compared to PLGA-based copolymers degradation results of our previously published article (pH 3.1 in 16 days)^6^, the upper pH value in the similar time interval is because of the lesser carboxylic acid groups in new copolymer maleate block.

FTIR analysis was used for investigation of the variation in structure of copolymer during the degradation process (refer to Figs. S7 and S8 and detailed explanation in the supplementary file). The degradation results showed that until 30 days, the degradation of copolymer was not evident, but after that the copolymer started the main process of degradation, due to the higher LA / GL ratio in PLGA section. This result is in agreement with other reports about PLGA based copolymers^27,30^.

### CMC results, characterization, encapsulation and loading efficiency of micelles

**Figure 4:**
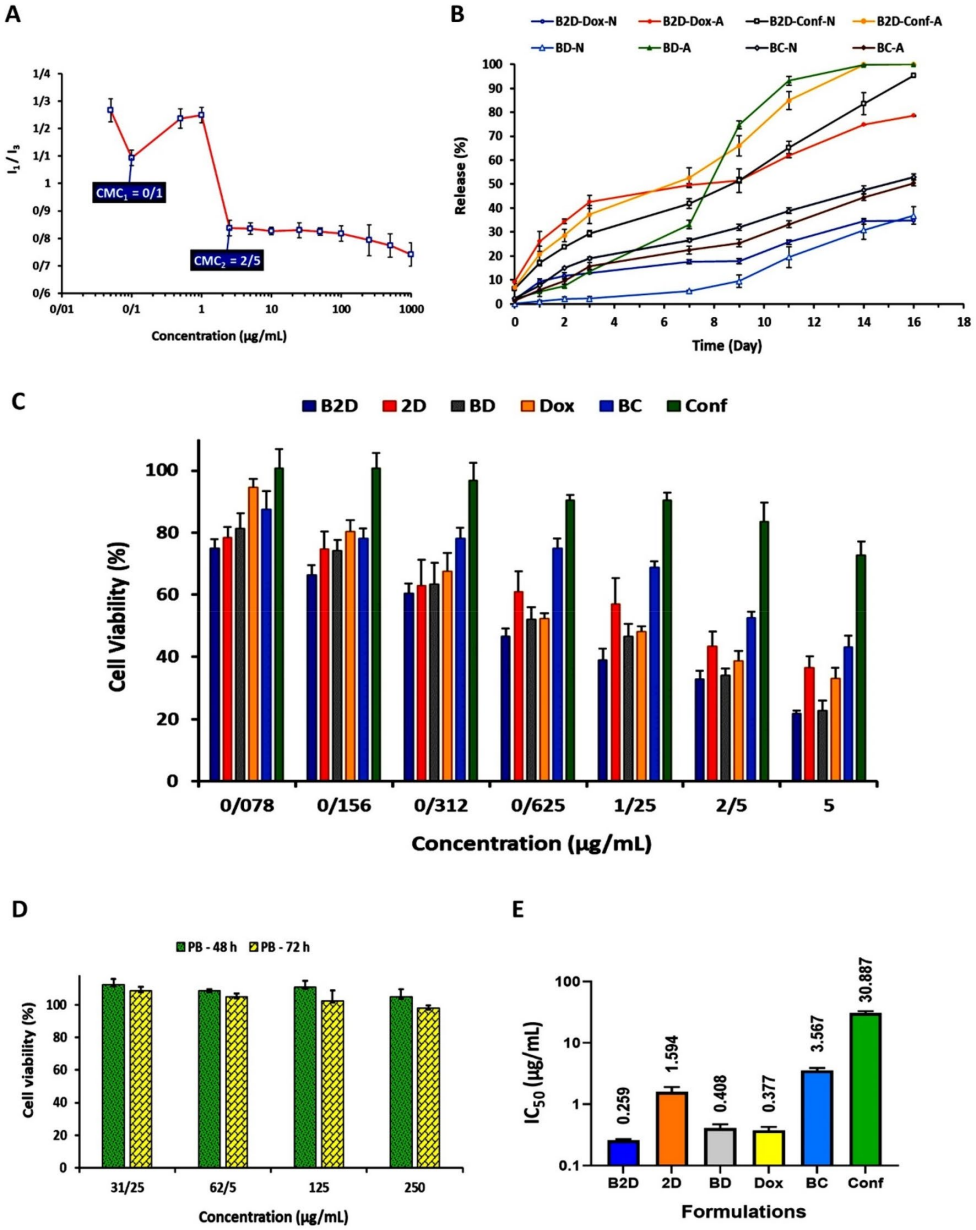
(**A**) Diagram of I_1_/I_3_ (I_373 nm_/I_393 nm_) versus βCD-g-PMA-co-PLGA copolymer concentration for determining of CMC values of copolymer, by spectrofluorometric method (copolymer concentration of: 0.05, 0.1, 0.5, 1, 2.5, 5, 10, 25, 50, 100, 250, 500, 1000 μg/mL, and final concentration of Pyrene: 0.005 μg/mL), n = 2; (**B**) Plot of release (%) of drugs from drug loaded nano formulations (**B2D**, **BD**, **BC**) versus time (day) (1 mg of nano formulations were dispersed in 2 mL of sink solution with pH 5.5 and 7.4 for various time intervals: 1, 2, 3, 7, 9, 11, 14 and 16 days), n = 2; (**C**) Enhanced anticancer efficacy by co-delivery of Dox and Conf in nano-formulation.The MDA-MB-231 cells viability (%) in the existence of formulations (**B2D**, **2D**, **BD**, **Dox**, **BC** and **Conf** with different concentrations: 0.078, 0.156, 0.312, 0.625, 1.25, 2.5 and 5 μg/mL), after 48 h, obtained from MTT method; (**D**) The MDA-MB-231 cells viability (%) in the presence of the blank βCD-g-PMA-co-PLGA micelles (PB) with various concentrations: 31.25, 62.5, 125, 250 μg/mL, after 48 and 72 h, by MTT method; (**E**) Diagram of IC_50_ dosage (μg/mL) of formulations (**B2D**, **2D**, **BD**, **Dox**, **BC** and **Conf**) calculated by GraphPad Prism software using MTT results of MDA-MB-231 cells after 48 h. The MTT results were analyzed statistically by GraphPad Prism software (n = 3, p < 0.05). (Abbreviations: **PB**: blank βCD-g-PMA-co-PLGA micelles, **B2D**: co-drug loaded βCD-g-PMA-co-PLGA micelles, **BD**: Dox loaded βCD-g-PMA-co-PLGA micelles, **BC**: Conferone loaded βCD-g-PMA-co-PLGA micelles, **Dox**: Free Doxorubicin, **Conf**: Free Conferone, **2D**: Free Doxorubicin-Conferone).

Critical micelle concentration (CMC) of copolymer was determined using a plot of concentration of pyrene loaded micellar solution versus the ratio of *I*_*1*_*/I*_*3*_ (Fig. 4A). With increasing of micelle formation, the pyrene loading in core of micelles increased and as a result the pyrene intensity decreased. After formation of micelles, the final value of a sharp decrease in the ratio of intensities is considered as CMC. As could be seen in Fig. 4A, the plot is "*µ-shaped*" with two minimum points that are selected as CMC_1_ and CMC_2_ for copolymer micellar solution. According to Fig. 4A, the first CMC point is located at 0.1 µg/mL and the second CMC point is observed at 2.5 µg/mL. Such type of CMC diagrams (*µ-shaped*) appears in copolymers micellization and is related to self-assembly process and polydispersity of copolymers (due to variation in chain length of polymeric blocks)^31^. It is also mentioned that with increasing of copolymer concentration, cylindrical-shaped micelles are formed as a result of aggregation of spherical-shaped micelles, which causes the second CMC^32^. The low value of CMC is an important and favorite property for dynamic stability of micelles, particularly at very low concentration in physiological environments such as blood circulation^33^. Our prepared *βCD-g-PMA-co-PLGA* micelles had a very low CMC (about 0.1 and 2.5 µg/mL) compared to what reported in the similar studies, and hence were in acceptable range for dynamic stability. For example, Qiu et al. reported that their β‑cyclodextrin-centered star-shaped amphiphilic polymers, had CMC about 2.3–38 µg/mL and 0.92–38 µg/mL^34,35^. Similarly, Lv et al. determined CMC values of about 0.98 and 52.4 µg/mL, for 6-armed and 3-armed βCD-based star copolymeric micelles^36^; Liu et al. obtained a CMC value of about 15 µg/mL, for their βCD-based star copolymeric micelles^37^. A study by Li et al. on mixed micelles (βCD-PLA-mPEG / TA-PLA-mPEG) showed a CMC value between 8.2 and 25.4 µg/mL^38^. Likewise, CMC of βCD-PELA micelles were determined by Ji et al. to be equal to 1.4 µg/mL^39^. Therefore, our newly developed micelles (β*CD-g-PMA-co-PLGA*) showed a CMC value of 9–520-fold smaller compared to CMC values for βCD-based micelles in the previously published reports.

Doxorubicin (**Dox**), Conferone (**Conf**) and combination of them (**2D**) were loaded to micelles as single and co-drug loaded β*CD-g-PMA-co-PLGA* micelles (**BD**, **BC** and **B2D**, respectively), with copolymer/drug ratio of 10:1. For confirming of **Dox** and **Conf** loading into micelles, FTIR spectrum of co-drug loaded β*CD-g-PMA-co-PLGA* micelles (**B2D**) was evaluated. According to Fig. S1-**B2D**, the presence of strong and broad peak at 1400–1500 cm^−1^ and 1650 cm^−1^ shows the stretching of **C**=**C** of Dox and Conf aromatic and alkene rings, respectively. Presence of =**C-H** in Dox and Conf proved by appearance of peak at 3100 cm^−1^. Peaks are observed at: 700 cm^−1^ (out of plane bending of **C–H** of aromatic ring) and at 1423 cm^−1^ (stretching of **C–C** band of aromatic ring) was indicators for presence of Dox-Conf in nano-formulation.

Drug loading results of nano-formulations are presented in Table 2 as drug encapsulation efficiency (*DEE %*). The high values of *DEE %* (up to 98%) in Table 2, show that the copolymeric micelles have very great loading efficiency, due to presence of various drug trapping positions (binding electrostatically to **–COO–** groups of PMA section, βCD cavity and core of micelle). Our obtained *DEE %* shows a very higher efficiency compared to similar studies on βCD-based star micelles with a range of 21.44–86.4%^34–36,40^.

**Table 2:** Results of drug encapsulation efficiency (DEE %) for nano-formulations (Abbreviations: B2D: co-drug loaded βCD-g-PMA-co-PLGA micelles, BD: Dox loaded βCD-g-PMA-co-PLGA micelles, BC: Conf loaded βCD-g-PMA-co-PLGA micelles).

Nano-formulations	B2D	BD	BC
Dox (*DEE %*)	99.50	98.65	–
Conf (*DEE %*)	99.99	–	99.93

The blank and co-drug loaded β*CD-g-PMA-co-PLGA* micelles were analyzed with DLS-zeta test and results are presented in Figs. S9-a, S9-b and S10. According to Fig. S9, zeta-potential of blank and co-drug loaded β*CD-g-PMA-co-PLGA* micelles are equal to − 19.7 and − 2.39 mV, respectively. This difference between zeta-potential of blank and co-drug loaded β*CD-g-PMA-co-PLGA* micelles is due to the electrostatic interactions between carboxylic acid groups of micelle surfaces (pK_a_ = 6.6) and **Dox** amine groups (pK_a_ = 8.3) at pH = 7.4. Decreasing of zeta-potential (from − 19.7 to − 2.39 mV) after drug loading, confirms loading of **Dox** on surfaces of micelles. However, **Dox** could be loaded into core of micelles, too. In the case of **Conf**, due to the high hydrophobicity, loading happens into the core of micelles completely. According to the published researches, the optimum range of zeta-potential for electrostatically stability and extended circulation time for nano-particles in blood, is ± 20 mV^41^. Therefore, the obtained zeta-potential values for blank and co-drug loaded β*CD-g-PMA-co-PLGA* micelles are located in the suitable range that complies with other related reports^42^.

Based on DLS results in Fig. S10, the blank β*CD-g-PMA-co-PLGA* micelles had an average hydrodynamic diameter of about 96.51 nm (with polydispersity index, PDI = 1). The obtained PDI value shows lower homogeneity of nano micelles^43,44^, that may be due to variation in amount of grafted-βCD, length of PLGA or PMA chains in copolymer. The size and morphology of blank β*CD-g-PMA-co-PLGA* micelles are analyzed with SEM and the prepared image is shown in Fig. S11. According to SEM results, the blank micelles had an average diameter of about 34.5 nm and a spherical-like shape. The DLS reported size is higher compared to what SEM reported, that is probably due to the swelling of micelles by water in DLS test versus the dry condition in SEM analysis^45^.

Altogether, based on the obtained desirable diameter and zeta-potential of the micelles, it can be claimed that the prepared micelles are capable of penetrating into cancer tissues and cells through passive targeting. On top of that, the prepared micelles had a smaller size in comparison with other βCD-based micelles in the published works up to now which improves the efficiency of diffusion into cells^34,35,46^.

### Investigation of in-vitro release test

**Dox** and/or **Conf** release from single- and co-drug loaded β*CD-g-PMA-co-PLGA* micelles, are shown in Fig. 4B. As can be seen, co-drug loaded and Dox loaded β*CD-g-PMA-co-PLGA* micelles showed pH-responsive release with more dominant release at pH = 5.5 compared to pH = 7.4. But conferone release from conferone loaded β*CD-g-PMA-co-PLGA* micelles did not follow the pH-responsive pattern. The reason for lower Dox release from **Dox**-loaded nano-formulations in physiological pH (pH = 7.4) was the presence of electrostatic interaction between protonated amine group of **Dox** (with positive charge) (pH < pKa = 8.3) and carboxylate groups of copolymer (with negative charge) (pH > pKa micelles = 6.6). While, in acidic pH (pH = 5.4), the carboxylate groups of copolymer are protonated (pH < pKa = 6.6) and transferred to **–COOH** group without any charge. Therefore, the electrostatic interaction between **Dox** and carboxylate part of micelle was removed that led to higher amount of **Dox** release. More importantly, **Dox** release from co-drug- and Dox loaded β*CD-g-PMA-co-PLGA* micelles has two steps (from day-1 to day-7, and from day-7 to day-16), that is probably due to the presence of more than one loading mechanisms. Since **Dox** could be loaded either in core of micelle or interact with carboxylate groups on the surface of micelles and finally interact with βCD as inclusion-complex, the release profile could be different depending on the loading mechanism.

In the case of **Conf,** it could just be loaded in the core of micelles or trapped in βCD cavity, due to the high hydrophobicity. Therefore, its release depends on the micelle's deformation (with no pH-sensitivity) which increases with copolymer degradation or micelles swelling. The dual drug release in our study is clearly sustained compared with Dox release from β*CD* based micelles published previously^34,35,47,48^. For example, in a study by Xu et al., βCD-PLA-POEGMA/Dox micelles had shown a sustained Dox release of about 20% and 50%, at pH = 7.4 and 5.0, respectively after 24 h^40^. In the same time, in our study, Dox and Conf release from drug loaded β*CD-g-PMA-co-PLGA* micelles was below 10% and 30%, at pH = 7.4 and 5.0, respectively. Sustained release of Dox in our work, may be due to the stability and rigidity of micelle structure and dominant loading of drugs in the core of micelles that cause a resistance against dilution and drug release.

### Cell internalization ability of micelles

**Figure 5:**
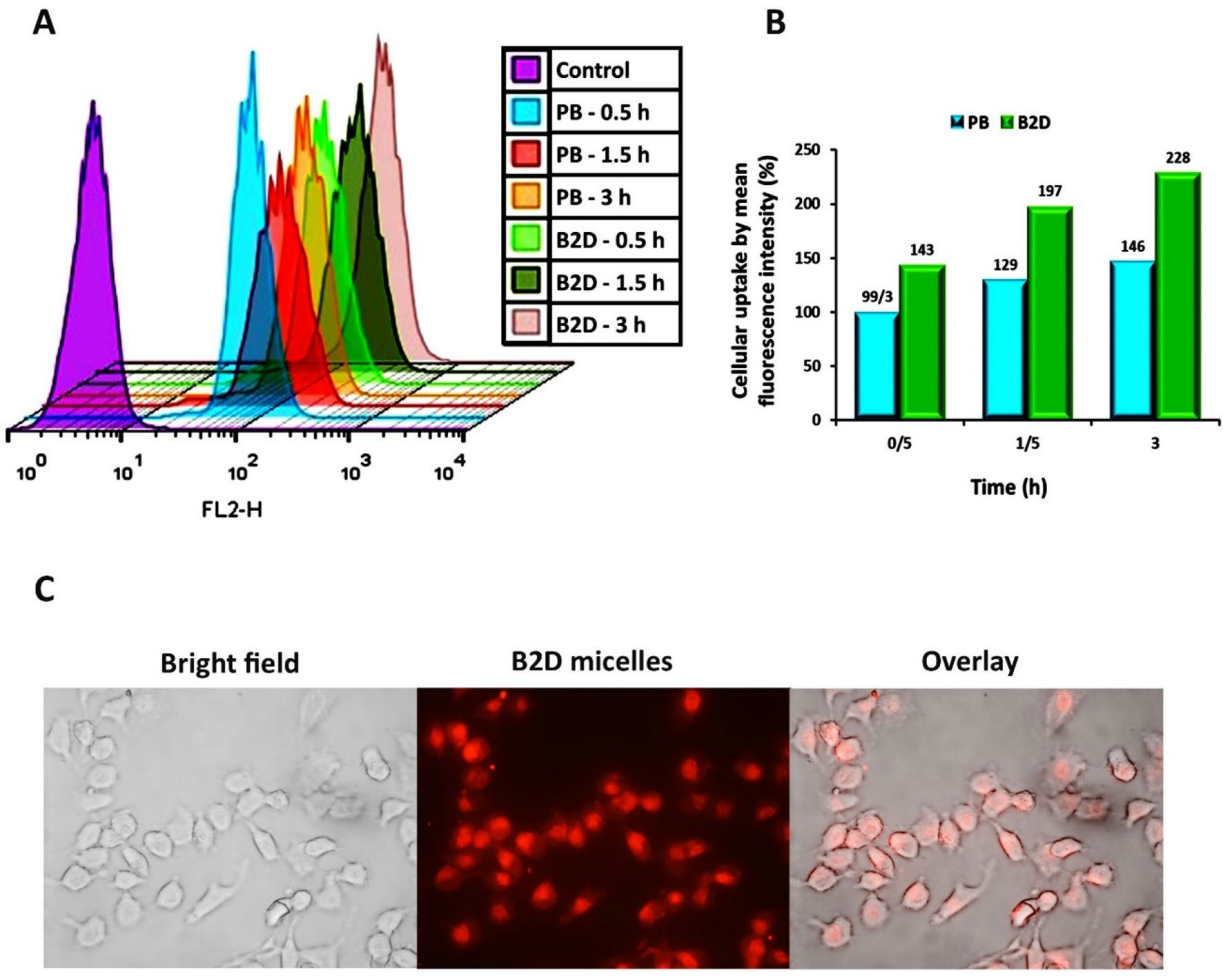
(**A**) Results of intracellular uptake of rhodamine B-labeled blank βCD-g-PMA-co-PLGA micelles (**PB**: 20 µg/mL) and rhodamine B-labeled co-drug loaded βCD-g-PMA-co-PLGA micelles (**B2D**: 2 µg/mL) by MDA-MB-231 cells in different time intervals: 0.5, 1.5 and 3 h, using flowcytometry; (**B**) Diagram of mean fluorescence intensity (%) of rhodamine B-labeled **PB** and **B2D** that were uptaken by MDA-MB-231 cells in different time intervals: 0.5, 1.5 and 3 h, using flowcytometry; (the differences between treatments was statistically significant, p < 0.001); (**C**) Fluorescence microscopic images of rhodamine B-labeled **B2D** (2 µg/mL) internalization to MDA-MB-231 cells only at 1 h, prepared by research fluorescence microscope. (Abbreviations: **PB**: Rhodamine B-labeled blank βCD-g-PMA-co-PLGA micelles, **B2D**: Rhodamine B-labeled co-drug loaded βCD-g-PMA-co-PLGA micelles).

Internalization of rhodamine B-labeled blank β*CD-g-PMA-co-PLGA* micelles (**PB**) and rhodamine B-labeled co-drug loaded β*CD-g-PMA-co-PLGA* micelles (**B2D**) into MDA-MB-231 cell line were investigated with flowcytometry and fluorescent microscope and the obtained results are presented in Figs. 5A–C. As shown in Fig. 5A, 100% of the cells have taken the rhodamine B-labelled blank β*CD-g-PMA-co-PLGA* micelles and rhodamine B-labeled co-drug loaded β*CD-g-PMA-co-PLGA* micelles. According to Fig. 5B, the mean fluorescence intensity (%) was increased with time (0.5, 1.5 and 3 h). Similarly, rhodamine B-labeled co-drug loaded β*CD-g-PMA-co-PLGA* micelles showed higher mean fluorescence intensity compared to rhodamine B-labeled blank β*CD-g-PMA-co-PLGA* micelles in all mentioned time intervals (p_value_ < 0.001). The higher cellular uptake of rhodamine B-labeled co-drug loaded co-drug loaded β*CD-g-PMA-co-PLGA* micelles compares to rhodamine B-labeled blank β*CD-g-PMA-co-PLGA* micelles is due to the decrease in the negative surface charge of co-drug loaded β*CD-g-PMA-co-PLGA* (− 2.39 mV) micelles compared to blank β*CD-g-PMA-co-PLGA* (− 19.7 mV) micelles as determined by the zeta potential, because the lower negative charge has less electrostatic repulsion forces with the negative cell membrane, and therefore higher uptake into the cells^49^.

Based on the rapid and great uptake percentage of our novel developed β*CD-g-PMA-co-PLGA* micelles (100% at 0.5 h), we can claim that the prepared β*CD-g-PMA-co-PLGA* micelles had favorable structure, charge, and size for cell internalization. Superiority of this formulation is clear when comparing its cell internalization with reports of other researchers. For example, our previous work showed a lower internalization of functionalized PLGA-based blank micelles into MDA-MB-231 cells (33%, 60 and 81%, at 0.5, 1.5 and 3 h) which is very slower^6^. This phenomenon is because of lesser negative charge of blank β*CD-g-PMA-co-PLGA* micelles in the present study (− 19.7 mV) compared to the blank micelle charge in previous study (− 29.7 mV). This leads to inferior electrostatic repulsion forces between negative charges of blank β*CD-g-PMA-co-PLGA* micelles and cell membrane and consequently higher internalization into cells^49^. Recent research reports show that blank and co-drug loaded β*CD-g-PMA-co-PLGA* micelles have faster cell internalization compared to other βCD-based nano particles published previously. For example, Pooresmaeil et al. reported that their blank and Dox loaded βCD-functionalized PAMAM dendrimers internalized near 100% into MDA-MB-231 cells after 3 h^50^. Niu et al. reported that their Dox/Melatonin loaded βCD containing functionalized graphene-dendrimeric system could be internalized up to 73.99 and 94.28% into Saos-2 cells after 2 and 4 h, respectively^51^.

Figure 5C shows the fluorescent microscopic images of MDA-MB-231 cells that uptook the rhodamine B-labeled co-drug loaded β*CD-g-PMA-co-PLGA* micelles.

### Cytotoxicity of drug loaded micelles

The MDA-MB-231 cells viability in the presence of all formulations (**PB**, **B2D**, **2D**, **BD**, **Dox**, **BC**, **Conf**) with different concentrations were investigated by MTT assay and the obtained results were presented in Fig. 4C,D. According to Fig. 4D, the blank β*CD-g-PMA-co-PLGA* micelles did not show any noticeable cytotoxicity on MDA-MB-231 cells in the studied concentration range (31.25, 62.5, 125, 250 μg/mL) after 48 and 72 h. Figure 4C shows that the single- and co-drug loaded β*CD-g-PMA-co-PLGA* micelles caused a higher level of cytotoxicity in comparison with the corresponding free drugs (**Dox**, **Conf** and **Dox-Conf**). This difference between result of nano-formulation and free drugs, was due to the higher intracellular uptake that overcome drug resistance as well as increasing of **Conf** solubility in micelle forms. GraphPad prism software (V. 8.0.1) was used to calculate *IC*_*50*_ dosages of all formulations; and the results are presented in Fig. 4E and Table S3. The results of cell viability assay showed that the cells treated with dual drug loaded (Dox-Conf loaded β*CD-g-PMA-co-PLGA*) micelles caused in significantly lower viability than those treated with either single drug loaded micelles, indicating that combination of Dox and Conf demonstrated superior anticancer activity. This result suggesting that efficient delivery of Dox and Conf by β*CD-g-PMA-co-PLGA* micelles contributes substantially to enhance combinational antitumor Effects (Fig. 4C,E). According to *IC*_*50*_ results, the lowest *IC*_*50*_ (0.259 μg/mL) belongs to co-drug loaded β*CD-g-PMA-co-PLGA* micelles. The effective dosage of **Dox** in co-drug loaded β*CD-g-PMA-co-PLGA* micelles (0.1295 μg/mL) was lower compared to free **Dox** and Dox loaded β*CD-g-PMA-co-PLGA* micelles (refer to Table S3) because conferone in combination with Dox, can overcome the P-gp-mediated drug resistance and lead to Dox accumulation in cells^52^. This caused a decrease in the required **Dox** therapeutic dosage and therefore a decrease in its side effects. Based on our literature review our novel developed co-drug loaded micelle showed superior anticancer efficacy compared to previously published articles. The *IC*_*50*_ values of co-drug- and Dox loaded β*CD-g-PMA-co-PLGA* micelles (0.259 and 0.408 μg/mL, respectively), are lower in comparison with the previous reports on Dox-loaded micelles. For example, Xu et al. reported an *IC*_*50*_ of about 10 μg/mL for Dox on HeLa cells, and Qiu et al. reported an *IC*_*50*_ of about 2 and 15 μg/mL of Dox, for MCF-7 and MCF-7/ADR cells, respectively^34,40^.

The CompuSyn software (V. 1) was used for calculation of combination index (*CI*), and results are shown in Fig. S12 and Table S4. The combination of free **Dox**-**Conf** and **Dox**-**Conf** in co-drug loaded β*CD-g-PMA-co-PLGA* micelles showed synergistic effects in *IC*_*50*_dosage (*CI* < 1). The *CI* value of co-drug loaded β*CD-g-PMA-co-PLGA* micelles (0.5) is lower than free **Dox**-**Conf** (0.8), that shows a more synergistic effect of nano-formulated combination form (co-drug loaded β*CD-g-PMA-co-PLGA* micelles). As can be seen in Fig. 4C, among the nano-formulations, the co-drug loaded β*CD-g-PMA-co-PLGA* micelles showed higher cytotoxicity in comparison with single-drug loaded β*CD-g-PMA-co-PLGA* micelles that could be explained by lower *IC*_*50*_ dose and synergistic effect. Drug efflux due to increasing in P-glycoprotein (P-gp) expression, is an important problem in progressive cancers which causes a decrease in drug accumulation in cells and hence a decrease in drug efficiency. Conferone in combination with **Dox**, can overcome the P-gp-mediated drug resistance and lead to **Dox** accumulation in cells^52^. As a result, co-drug loaded β*CD-g-PMA-co-PLGA* micelles acted as the most efficient nano-formulation because of higher accumulation level of **Dox**, increasing of **Conf** solubility, and synergistic effect. The statistical analysis showed that the results of comparison among groups was significant.

### Evaluation of cell cycle arrest induced by drug loaded micelles

**Figure 6:**
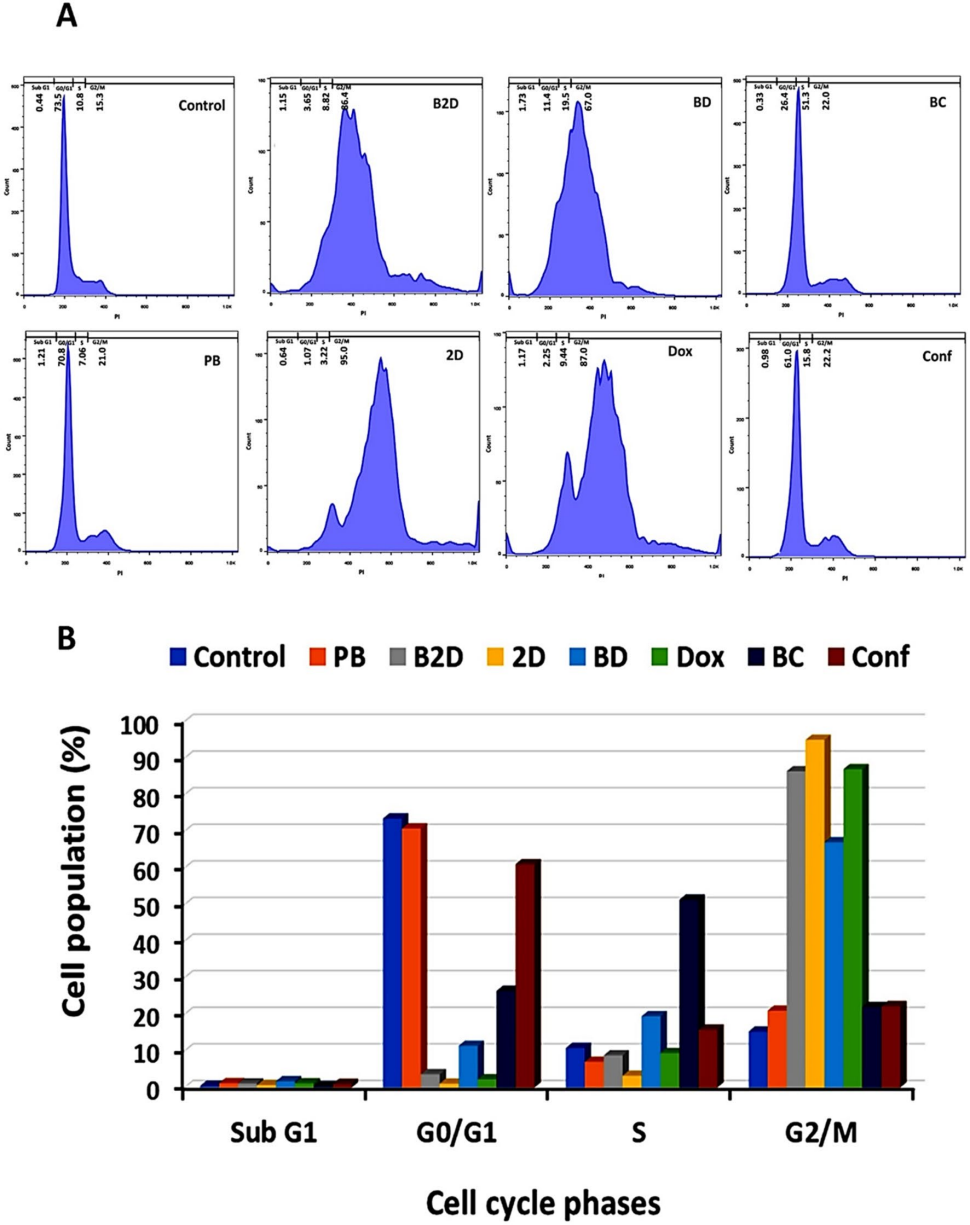
Results of cell cycle test of MDA-MB-231 cells treated with all formulations (0.259 μg/mL of **B2D**, **2D**, **BD**, **Dox**, **BC** and **Conf** and 2.59 μg/mL of **PB**) by flowcytometry method: (**A**) Histograms of cell cycle distrubution and (**B**) Quantitative diagram of cell population (%) in different phases of cell cycle (Sub G1, G0/G1, S and G2/M), in presence of all formulations (Abbreviations: **PB**: blank βCD-g-PMA-co-PLGA micelles, **B2D**: co-drug loaded βCD-g-PMA-co-PLGA micelles, **BD**: Dox loaded βCD-g-PMA-co-PLGA micelles, **BC**: Conferone loaded βCD-g-PMA-co-PLGA micelles, **Dox**: Free Doxorubicin, **Conf**: Free Conferone, **2D**: Free Doxorubicin-Conferone).

The cell cycle analysis investigates the various stages of cell cycle and DNA duplication, containing: G1, S, G2 and M^53^. The obtained results are presented in Fig. 6 and Table S5.

According to Fig. 6 and Table S5, the blank β*CD-g-PMA-co-PLGA* micelles did not show noticeable changes in cell cycle pattern in comparison with the control group, which shows almost no toxicity to MDA-MB-231 cells. The co-drug loaded β*CD-g-PMA-co-PLGA* micelles (G2/M: 86.4%), free Dox-Conf combination (G2/M: 95%), and free Dox caused (G2/M: 87%), G2/M arrests in treated cells (0.259 μg/mL) while, Dox loaded β*CD-g-PMA-co-PLGA* micelles showed S (19.5%) and G2/M (67%) arrest compared to control group (S: 10.80%, G2/M = 15.30%) (Table S5). The presence of Conf in formulations lead to S arrest in Conf loaded β*CD-g-PMA-co-PLGA* micelles (S: 51.3%) and free Conf (S: 15.8%) (Table S5). S and G2/M arrests are the signs of strong inhibition to DNA duplication and can be seen in apoptosis. Pooresmaeil et al. showed that Dox loaded βCD-functionalized PAMAM dendrimers caused sub G1 (60%) arrest in MDA-MB-231 cells^50^. There are many reports that show Dox-Adjuvant combination therapy leads to sub G1, S and G2/M arrest in different cell lines. For example, Sabzichi et al. reported that Dox in combination with Quinacrine (QC) caused G2/M arrest (39% in 2.5 μM + 1.2 μM dosage of QC + DOX) in MDA-MB-231 cells^4^. Our co-drug loaded β*CD-g-PMA-co-PLGA* micelles caused a higher level of G2/M arrest (86.4%) in lower *IC*_*50*_ dosage of combination form (0.22 μM **Dox** + 0.34 μM **Conf**). Therefore, the combination of **Conf** with **Dox** had a higher synergistic effect on MDA-MB-231 cells, in comparison with combination of QC-Dox. Dox-Conf loaded micelles in our previous study^6^ showed sub G1, S and G2/M arrest which is in agreement with the current study. Similarly, Sabzi et al. showed that Dox and curcumin loaded micelles cause a sub G1 arrest in MDA-MB-231 cells^3^. In another study, Ahmadi et al. showed that Dox and hydroxytyrosol loaded micelles lead to sub G1 and S arrest in HT29 cells^54^. Rahimi et al. reported that combination of Dox and methotrexate on chitosan-based dendrimers lead to G2/M arrest in MCF-7 cells^55^. In our study, the conferone loaded β*CD-g-PMA-co-PLGA* micelles and free **Conf** lead to S arrest. Cheraghi et al. reported that conferone cause sub G1 and S arrest in HT-29 cell line with a time dependent manner^7^ which is in agreement with our results. The higher levels of S arrest in conferone loaded β*CD-g-PMA-co-PLGA* micelles (S: 51.3%), compared to the free **Conf** (S: 15.8%), is due to the increasing of solubility and intracellular uptake of **Conf** in conferone loaded β*CD-g-PMA-co-PLGA* micelles. The high levels of cell arrests for nano formulation-treated cells, show higher apoptosis of cells due to high cell internalization^56,57^.

### Evaluation of apoptosis induction

**Figure 7:**
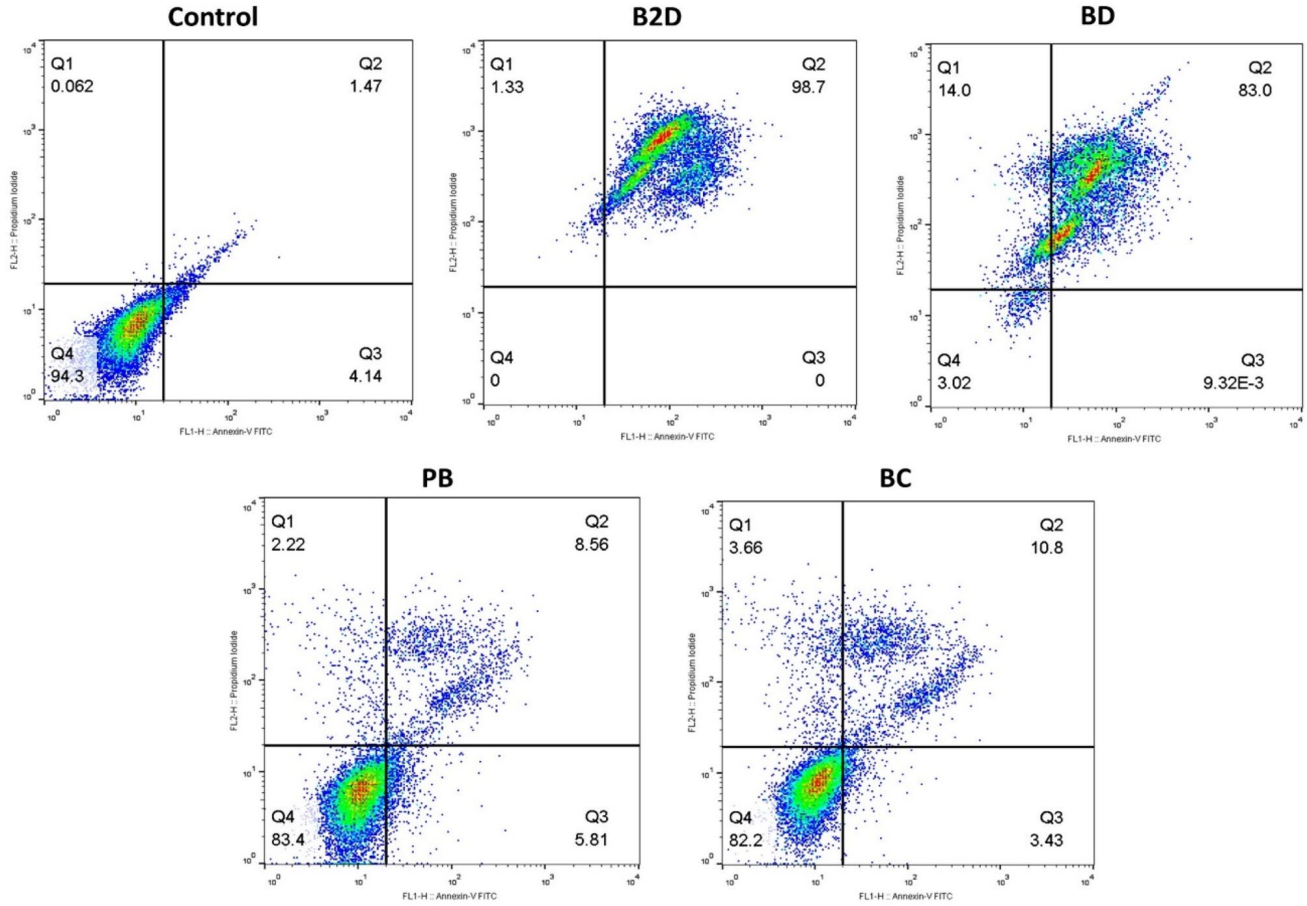
Enhanced antitumor effecacy by increased apoptotic cell death induced by co-delivery of Dox and Conf nano-formulation. Results of apoptosis test of MDA-MB-231 cells treated by nano formulations (0.259 μg/mL of **B2D**, **BD**, **BC** and 2.59 μg/mL of **PB**) obtained by flowcytometry method. (Abbreviations: **PB**: blank βCD-g-PMA-co-PLGA micelles, **B2D**: co-drug loaded βCD-g-PMA-co-PLGA micelles, **BD**: Dox loaded βCD-g-PMA-co-PLGA micelles, **BC**: Conferone loaded βCD-g-PMA-co-PLGA micelles).

Annexin-V is a fluorescent agent that stained the apoptotic cells and propidium iodide (PI) was used for staining nucleus of late apoptotic and necrotic cells^58^. To demonstrate that Dox-Conf loaded β*CD-g-PMA-co-PLGA* micelles produces greater level of cancer cell apoptosis compared to single drug loaded formulations and free drugs, MDA-MB-231 cells after treatment were analyzed by Annexin-V/PI double staining flowcytometry. The effect of free drugs (**Dox**, **Conf** and **Dox-Conf**) on apoptosis of MDA-MB-231 cells were presented in our previously published paper^6^. Figure 7 and Table S6, show the outcomes of apoptosis analysis. As could be seen in Fig. 7, the blank β*CD-g-PMA-co-PLGA* micelles did not show noticeable toxic effect (83.4% cell viability) to MDA-MB-231 cells. The Conf loaded β*CD-g-PMA-co-PLGA* micelles (**BC**) was not shown noticeable apoptosis and its result was similar to the result of blank β*CD-g-PMA-co-PLGA* micelles (**PB**). Because *IC*_*50*_ dosage (0.259 μg/mL) of co-drug loaded β*CD-g-PMA-co-PLGA* micelles was selected for all drug-loaded micelles in this test which is much lower than *IC*_*50*_ dosage of Conf loaded β*CD-g-PMA-co-PLGA* micelles (3.567 μg/mL). According to the results the co-drug loaded β*CD-g-PMA-co-PLGA* micelles showed synergistic effect with highest apoptosis (98.7%) and lowest necrosis (1.33%) compared to single-drug (**Dox** or **Conf**) loaded β*CD-g-PMA-co-PLGA* micelles. These results confirm that Dox-Conf loaded micelle acts as an effective intracellular co-delivery system that enhances combinational apoptosis-inducing effect. As expected from the results of cell cycle and MTT tests, the highest anticancer effect was observed in co-drug loaded β*CD-g-PMA-co-PLGA* micelles. This is the consequence of synergistic effect of drugs in co-drug loaded β*CD-g-PMA-co-PLGA* micelles as well as promotive effect of Conf on Dox intracellular accumulation. Superiority of Dox-Conf loaded β*CD-g-PMA-co-PLGA* micelles is clear when comparing its ability in induction of apoptosis (98.7%) with reports of other researchers. Li et al. concluded that their targeted delivery of Dox and Bcl-2 siRNA by βCD and folic acid containing nanocomplexes (FA-HP-βCD-PEI/DOX/siRNA) caused about 70% apoptosis of MCF-7/ADR cells^59^. Niu et al. quantitatively assessed apoptosis in cancer cells by Dox and Melatonin loaded βCD-containing nanoparticles (Dox/MLT-NPs) and witnessed 53.52% and 41.81% apoptosis of MG-63 and Saos-2 cells, respectively^51^. Ji et al. reported that their Dox-loaded βCD-based micelles (PELA54-CD-Dox) induced about 84.8% apoptosis in HL60/ADR cells^39^. Sabzichi et al. reported 40% apoptosis in MDA-MB-231 cells treated with combination of Dox-Quinacrine^4^. Li et al. showed that their combination therapy by Dox-Oridonin caused 64.46% apoptotic and 18% necrotic death of MDA-MB-231 cells^60^. In study by Sabzi et al., combination therapy of MDA-MB-231 cell line with Dox-curcumin loaded micelles showed 96% apoptosis^3^. Rahmani et al. reported that Dox-Conferone loaded micelles led to 95% apoptosis in MDA-MB-231 cells^6^. According to Fan et al. Dox-Gamabufotalin loaded NPs induced about 79–89.2% apoptosis to MDA-MB-231 cells^61^. In comparison to the recent literature, our developed Dox-Conf loaded β*CD-g-PMA-co-PLGA* micelles showed superior performance in induction of apoptosis in MDA-MB-231 cells.

### Investigation of apoptosis pathway by real-time PCR

**Figure 8:**
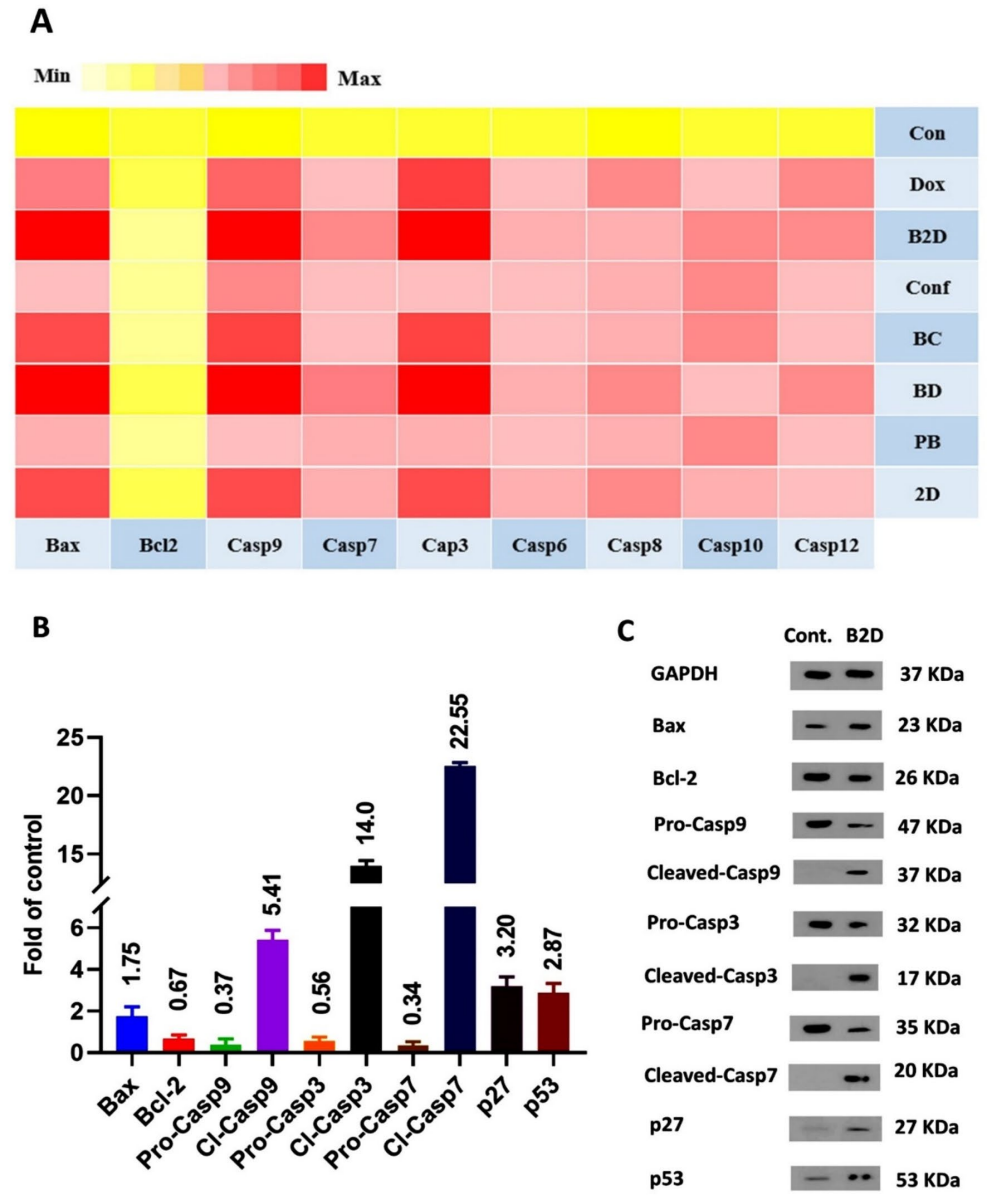
(**A**) Results of real-time PCR analysis of MDA-MB-231 cells treated with all formulations (0.259 μg/mL of **B2D**, **2D**, **BD**, **Dox**, **BC** and **Conf** and 2.59 μg/mL of **PB**) as the heat map of expression level of genes related to un-treated control group (gene expresion = 1). The yellow and light-yellow colors are the signs of lack of gene expression or downregulation of it and red color shows upregulation of gene expression related to the control (genes: Bcl-2, Bax, Caspase-3, Caspase-6, Caspase-7, Caspase-8, Caspase-9, Caspase-10, Caspase-12 and GAPDH as the internal control gene); (**B**) Diagram of proteins expression changes related to the control group (protein expression = 1) obtained from western blotting (the MDA-MB-231 cells were treated by **B2D** with concentration of 0.259 μg/mL). Proteins: Bcl-2, Bax, pro-Caspase-9, Cleaved-Caspase-9, pro-Caspase-3, Cleaved-Caspase-3, pro-Caspase-7, Cleaved-Caspase-7, p27 and p53, and GAPDH as internal control, n = 2; (**C**) Images of western blotting of the MDA-MB-231 cells treated with **B2D** (0.259 μg/mL). Un-treated cells were considered as the control group. (Abbreviations: **PB**: blank βCD-g-PMA-co-PLGA micelles, **B2D**: co-drug loaded βCD-g-PMA-co-PLGA micelles, **BD**: Dox loaded βCD-g-PMA-co-PLGA micelles, **BC**: Conferone loaded βCD-g-PMA-co-PLGA micelles, **Dox**: Free Doxorubicin, **Conf**: Free Conferone, **2D**: Free Doxorubicin-Conferone).

With the aim of precise investigation of apoptosis pathway, the real-time PCR test was performed. The Bax and Bcl-2 proteins have pro-apoptotic and anti-apoptotic functions, respectively; and cytochrome-c production by mitochondria is controlled by these proteins. Simultaneous upregulation of Bax and downregulation of Bcl-2 expression leads to cytochrome-c release which consequently causes apoptosome formation. The apoptosome creation activates the caspase-9 and subsequently upregulation of caspase-9 which cause cleavage of effector caspase^62^. Basically, the cysteine protease enzymes (the caspases) are the essential factors for apoptosis and are divided into initiator (Caspase-8 and -9) and effector caspases (caspase-3 and -7)^56^. The caspase-8 and 12 upregulation are the signs of extrinsic pathway of apoptosis, but the caspase-9, caspase-3 and -7 upregulation show the intrinsic or mitochondria mediated pathway of cell apoptosis. Therefore, all the mentioned factors regulation changes after treating of cells with each formulation, which were investigated in real-time PCR analysis. The results of real-time PCR test were presented, as the heat map, of change in gene expressions related to the control group (gene expression = 1, Fig. 8A). In the heat map the light-yellow represented the lack of gene expression and red presented higher expression of genes. According to Fig. 8A, gene expression and regulation in blank β*CD-g-PMA-co-PLGA* micelles, compared to control group, did not show significant changes that confirmed its non-toxicity on MDA-MB-231 cells. Except for blank β*CD-g-PMA-co-PLGA* micelles, the rest of formulations showed concurrent Bax upregulation and Bcl-2 downregulation with the following order: co-drug loaded β*CD-g-PMA-co-PLGA* micelles > **Dox**-loaded β*CD-g-PMA-co-PLGA* micelles > free **Dox** ≈ **Conf**-loaded β*CD-g-PMA-co-PLGA* micelles ≈ free **Dox**-**Conf** > free **Conf**. The mentioned concurrent regulations cause upregulation of caspase-9 expression with the same order. As a result of caspase-9 upregulation, the caspase-3 and -7 were activated and caused cell apoptosis. Therefore, the Bax, caspase-9, caspase-3, and caspase-7 were upregulated dominantly while the Bcl-2, was downregulated significantly in our nano formulations (Co-drug loaded, **Dox**-loaded and **Conf**-loaded β*CD-g-PMA-co-PLGA* micelles). Based on the results, it can be concluded that the nano-formulations specially co-drug loaded β*CD-g-PMA-co-PLGA* micelles caused the higher level of cell apoptosis via caspase-dependent and intrinsic pathway of apoptosis. This superiority conforms with results of previous research about the effect of **Dox**-**Conf** loaded micelles on apoptosis pathway in MDA-MB-231 cell line^6^. Sabzi et al. showed that their Dox-Curcumin loaded micelles induced apoptosis to MDA-MB-231 cells via Bcl-2/Bax, caspase-9, caspase-7 and caspase-3 intrinsic pathway^3^. Moreover, Shafa et al. showed that apoptosis of DU145 prostate cancer cells in presence of combination of Dox with metformin was done via p21 and caspase-3 root^63^. Similarly, Khaki-khatibi et al. reported that according to RT-PCR results, their Dox-Stattic combination therapy caused BCl-2 downregulation and Bax upregulation in ZR-75–1 breast cancer cells^64^.

### Investigation of apoptosis pathway by western blotting

Since real-time PCR results showed that the highest level of caspase-dependent intrinsic pathway of apoptosis (at gene level) was induced by co-drug loaded β*CD-g-PMA-co-PLGA* micelles, the effect of co-drug loaded β*CD-g-PMA-co-PLGA* micelles on Bax, Bcl-2, pro-caspase-9, cleaved-caspase-9, pro-caspase-3, cleaved-caspase-3, pro-caspase-7, cleaved-caspase-7, p27 and p53 were evaluated using western blotting (at protein level). Cyclin dependent kinase inhibitor or KIP1 (p27) and the other tumor-suppressor proteins such as p53, are the cell cycle inhibitors. Upregulation of p27 and p53 cause Bax upregulation and Bcl-2 downregulation which lead to cell apoptotic death^65–67^. In the case of malignant tumors, the downregulated p53 prevents from apoptotic death^68^. Figure 8B,C and Table S7, present the western blotting results and fold-changes of protein expression in MDA-MB-231 cells treated by co-drug loaded β*CD-g-PMA-co-PLGA* micelles. These results show noticeable increase in expression of Bax (1.75-fold), cleaved-caspase-9 (5.41-fold), cleaved-caspase-3 (14-fold), cleaved-caspase-7 (22.55-fold), p27 (3.2-fold), p53 (2.87-fold), and decrease in expression of Bcl-2 (0.67-fold), pro-caspase-9 (0.37-fold), pro-caspase-3 (0.56-fold) and pro-caspase-7 (0.34-fold), with respect to the control group. The upregulated p27 and p53, induced upregulation of Bax and reduction of Bcl-2 which caused a severe disturbance to cell cycle, and subsequently cell apoptosis. Increasing in Bax and decrease in Bcl-2 expression led to cytochrome-c release from mitochondria, and hence creation of apoptosome which led to pro-caspase-9 expression. Upregulation of pro-caspase-9, caused cleavage of caspase-9 in parallel with pro-caspase-9 downregulation. Cleaved-caspase-9 upregulation caused pro-caspase-3 and pro-caspase-7 upregulation and their cleavage (upregulation of cleaved-caspase-3 and cleaved-caspase-7). Finally, cleavage of death substrate increased and hence fragmentation of DNA was occurred as a result of upregulation of cleaved-caspase-3 and cleaved-caspase-7. Because of marked increase in expression of cleaved-caspase-9, -3 and -7, it was proved that the co-drug loaded β*CD-g-PMA-co-PLGA* micelles induced apoptosis to MDA-MB-231 cells via intrinsic mitochondrial pathway (p27, p53, Bcl-2/Bax, cleaved-caspspase-9, cleaved-caspase-7 and cleaved-caspase-3 axis) which confirmed the real-time PCR outcomes. Similarly, Wei et al., reported that Dox in combination with Magnoflorine led to apoptosis of MDA-MB-231 cells via Bax/Bcl-2; cleaved-caspase-9 and cleaved-caspase-3 pathway^69^. Li et al. , using western blotting, reported that Dox-Oridonin combination induced apoptosis to MDA-MB-231 cells via Bcl-2/Bax, cleaved-caspase-3 and cleaved-PARP pathway^60^. Fan et al. showed that combination of Dox with Gamabufotalin induced apoptosis to MDA-MB-231 cells via p53, Bcl-2/Bax and cleaved-caspase-3 root^61^. Sabzi et al. reported that Dox-curcumin loaded micelles induced apoptosis to MDA-MB-231 cells via Bcl-2/Bax, cleaved-caspase-9, cleaved-caspase-7, cleaved-caspase-3 and p27^3^. In the same way, Rahmani et al. showed that Dox-conferone loaded micelles induced apoptosis via intrinsic Bcl-2/Bax, cleaved-caspase-9, cleaved-caspase-7, cleaved-caspase-3 and p27 pathway^6^. Therefore, it can be stated that Dox combination therapy on MDA-MB-231 cells induces apoptosis via activation of the intrinsic pathway. Our novel developed co-drug loaded β*CD-g-PMA-co-PLGA* micelles acted with similar intrinsic apoptosis pathway.

## Conclusion

The new pH-sensitive and biodegradable βCD-grafted poly maleate-block-PLGA micelles was developed for codelivery of Doxorubicin (**Dox**) and Conferone (**Conf**) into MDA-MB-231 cell line. Micelles with very low CMC (0.1 μg/mL), small size (34.5 nm) and negative zetapotential were obtained. The co-drug loaded β*CD-g-PMA-co-PLGA* micelles and **Dox** loaded β*CD-g-PMA-co-PLGA* micelles had a pH-sensitive and sustained drug release. The blank β*CD-g-PMA-co-PLGA* micelles and co-drug loaded β*CD-g-PMA-co-PLGA* micelles were internalized quickly (0.5 h) and completely (100%) into MDA-MB-231, because of their favorable size and zetapotential. The lowest *IC*_*50*_ (0.259 μg/mL) was obtained in B2D nano-formulation because of: synergistic effect of **Conf** on **Dox** (*CI* = 0.529), inhibition of P-gp expression and **Dox** efflux by **Conf** in MDA-MB-231 cells. Furthermore, co-drug loaded β*CD-g-PMA-co-PLGA* micelles with G2/M arrest, caused a severe disturbance to cell cycle and therefore induced exceptional apoptosis (up to 98%, according to cell cycle and apoptosis tests). The induced apoptosis of MDA-MB-231 cells by co-drug loaded β*CD-g-PMA-co-PLGA* micelles was confirmed with real-time PCR (at gene level) and western blotting (at protein level) that proved the p27, p53, Bax/Bcl-2; caspase-9; caspase-7 and caspase-3, intrinsic mitochondrial apoptosis pathway. The new **Dox**-**Conf** loaded β*CD-g-PMA-co-PLGA* micelles improved **Dox** therapeutic function by minimizing **Dox** therapeutic dosage. Thus, based on the excellent capabilities for apoptosis induction, β*CD-g-PMA-co-PLGA* micelles loaded with Dox in combination with Conf as adjuvant are suggested for in-vivo application in the future animal studies. We also aim to draw the attention of the scientific community to more consider the mechanisms involved in the synergism effect of combination therapy of anticancer drug and adjuvants with reduced side effects, and conduct clinical studies, for the development of alternative therapeutic way to benefit cancer patients worldwide.

## Supplementary Information

Supplementary Information.

## Abbreviations

β*CD**-g-PMA-co-PLGA*: β-Cyclodextrin grafted poly maleate-co-poly (lactide-co-glycolide)
β*CD**-g-PMA-OH*: β-Cyclodextrin grafted poly maleate
2D: Free doxorubicin-conferone
B2D: Co-drug loaded *βCD-g-PMA-co-PLGA* micelles
BC: Conferone loaded β*CD-g-PMA-co-PLGA* micelles
BD: Dox loaded β*CD-g-PMA-co-PLGA* micelles
Conf: Free conferone
Dox: Free doxorubicin
*MA*: Maleic anhydride
*ME*: 2-Mercapto ethanol
PB: Blank β*CD-g-PMA-co-PLGA* micelles
*PMA-OH*: Hydroxy terminated poly maleic anhydride
RB: Rhodamine B
