# Supplementary material for: Co-delivery of doxorubicin and conferone by novel pH-responsive β-cyclodextrin grafted micelles triggers apoptosis of metastatic human breast cancer cells
Source: Sci Rep. 2021 Nov 2;11:21425. doi: 10.1038/s41598-021-00954-8 (PMC8563731; doi:10.1038/s41598-021-00954-8)
Supplement: Supplementary file 1 — Supplementary Information. [file 41598_2021_954_MOESM1_ESM.docx]

Appendix 1: Supplementary information of:

**Co-delivery of doxorubicin and conferone by novel pH-responsive β-cyclodextrin grafted micelles triggers Apoptosis of metastatic human breast cancer cells**

Akram Rahmani ^a^, Fariborz Rahimi ^b^, Mehrdad Iranshahi ^c^, Houman Kahroba ^d, e^, Amir Zarebkohan ^f^, Mehdi Talebi ^g^, Roya Salehi ^h*^, Hassan Zavvar Mousavi ^i*^.

^a^ Department of Applied Chemistry, Faculty of Chemistry, Semnan University, Semnan, Iran

^b^ Department of Electrical Engineering, University of Bonab, Bonab, Iran

^c^ Faculty of Pharmacy, Mashhad University of Medical Sciences, Mashhad, Iran

^d^ Molecular Medicine Research Center, Biomedicine Institute, Tabriz University of Medical Sciences, Tabriz, Iran

^e^ Department of Molecular Medicine, Faculty of Advanced Medical Sciences, Tabriz University of Medical Sciences, Tabriz, Iran

^f^ Department of Medical Nanotechnology, Faculty of Advanced Medical Sciences, Tabriz University of Medical Sciences, Tabriz, Iran

^g^ Department of Applied Cell Science, Faculty of Advanced Medical Sciences, Tabriz University of Medical Sciences, Tabriz, Iran

^h^ Drug Applied Research Center and Department of Medical Nanotechnology, Faculty of Advanced Medical Sciences, Tabriz University of Medical Sciences, Tabriz, Iran. E-mail: [salehiro@tbzmed.ac.ir](mailto:salehiro@tbzmed.ac.ir)

^I^ Department of Chemistry, Faculty of Science, University of Guilan, P.O. Box 41335-1914, Rasht, Iran. E-mail: [hzmousavi@guilan.ac.ir](mailto:hzmousavi@guilan.ac.ir)

Table S1: Sequences of the primers in Real Time-PCR test

| Gene | Forward primer (5'-3') | Reverse primer (5'-3') |
| --- | --- | --- |
| CASPASE-3 | GAAATTGTGGAATTGATGCGTGA | CTACAACGATCCCCTCTGAAAAA |
| CASPASE-6 | ATGGCGAAGGCAATCACATTT | GTGCTGGTTTCCCCGACAT |
| CASPASE-7 | AGGGACCGAGCTTGATGATG | CACTGGGATCTTGTATCGAGGA |
| CASPASE-8 | GATCAAGCCCCACGATGAC | CCTGTCCATCAGTGCCATAG |
| CASPASE-9 | CTTCGTTTCTGCGAACTAACAGG | GCACCACTGGGGTAAGGTTT |
| CASPASE-10 | AGAAACCTGCTCTACGAACTGT | GGGAAGCGAGTCTTTCAGAAG |
| CASPASE-12 | TGTTACAAAGGCTCATGTGGAAA | GGGTCAGTATATTTGGGGTCTCA |
| Bax | TTCTGACGGCAACTTCAACT | CAGCCCATGATGGTTCTGAT |
| Bcl-2 | GGGAATCGATCTGGAAATCCTC | GGCAACGATCCCATCAATCT |
| GAPDH | ACAACTTTGGTATCGTGGAAGG | GCCATCACGCCACAGTTTC |


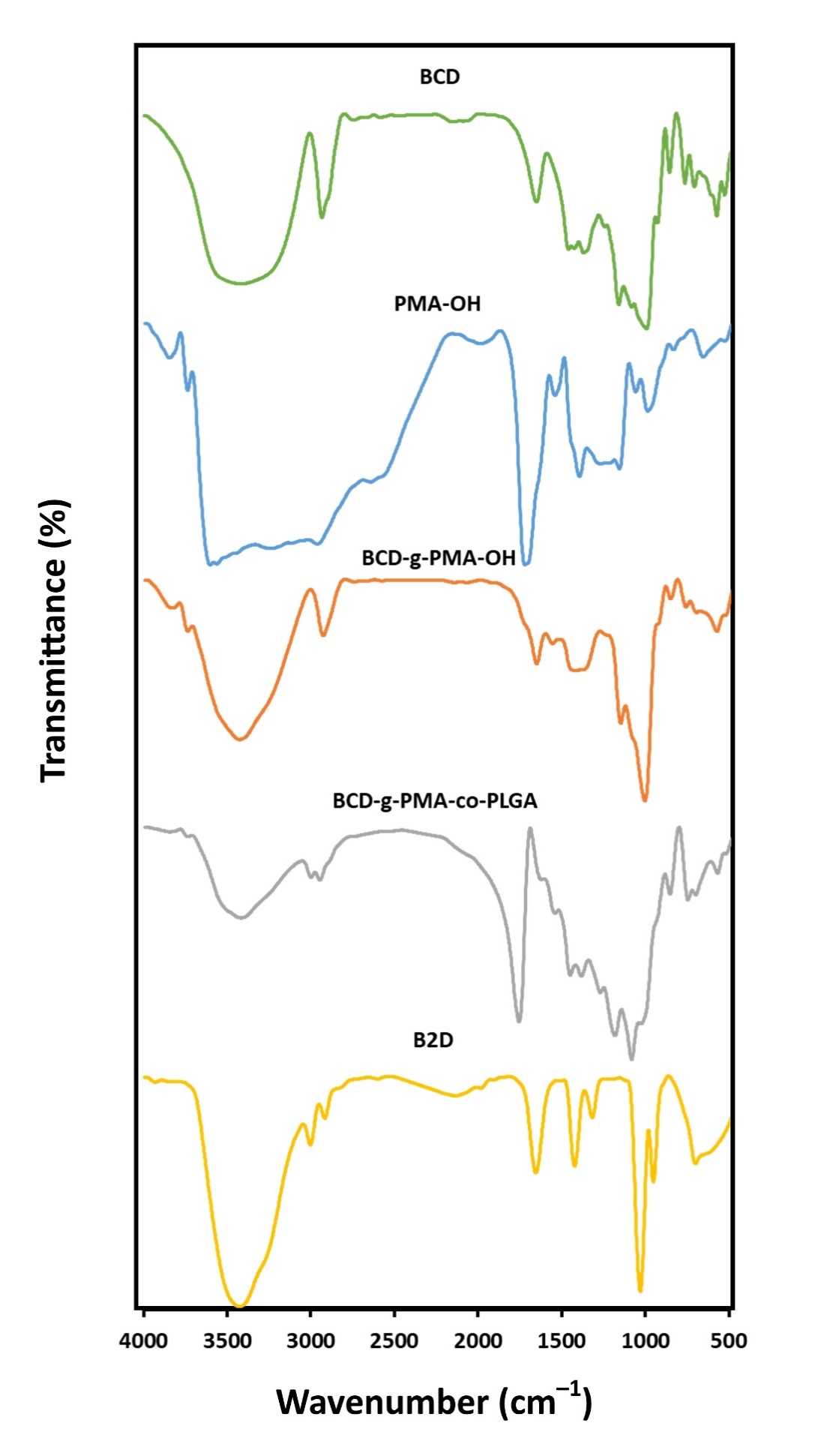


Figure S1: FTIR spectra of βCD, PMA-OH, βCD-g-PMA-OH, βCD-g-PMA-co-PLGA and co-drug loaded βCD-g-PMA-co-PLGA micelles (**B2D**).


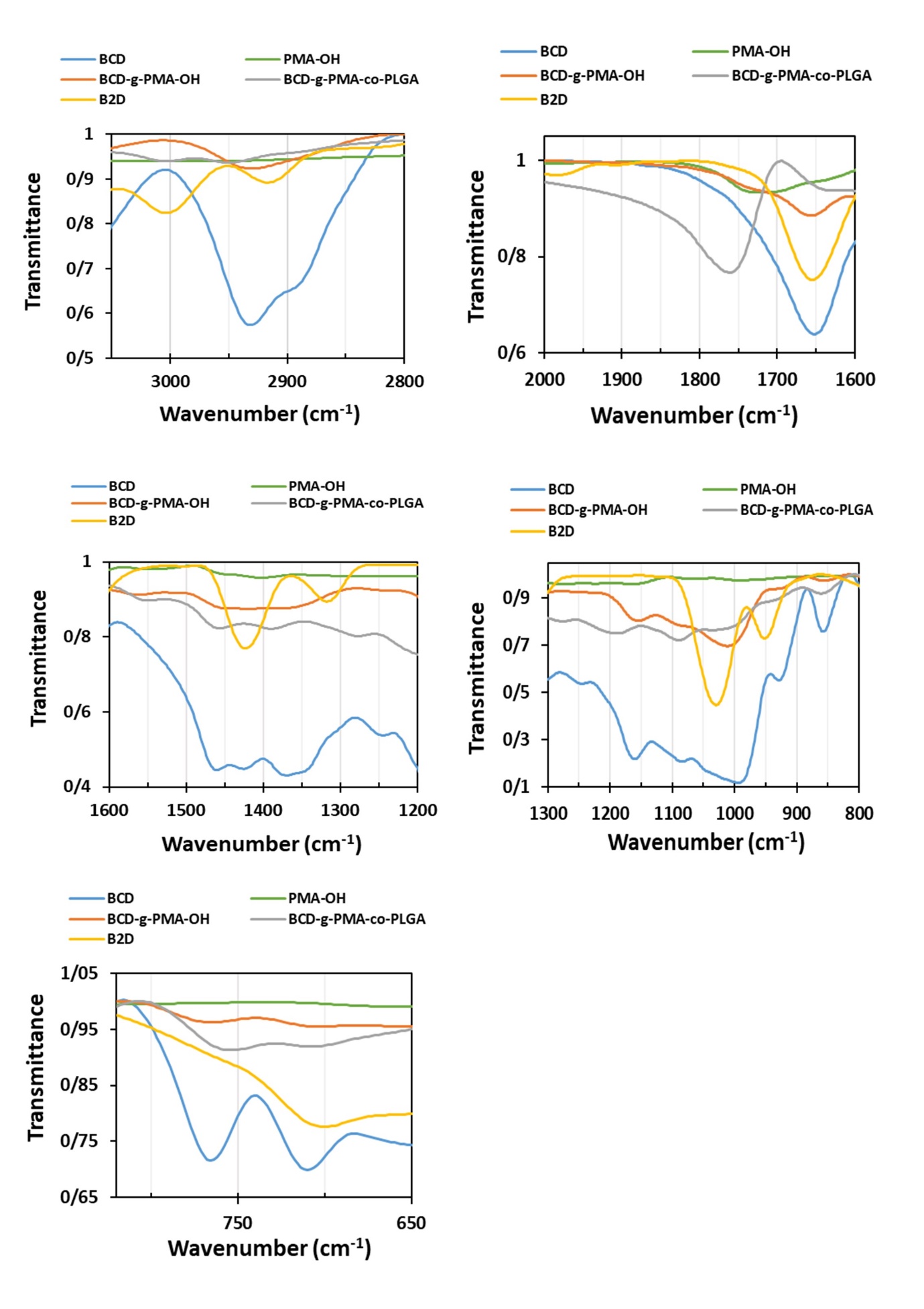


Figure S2: Enlarged FTIR spectra of βCD, PMA-OH, βCD-g-PMA-OH, βCD-g-PMA-co-PLGA and **B2D**

**FTIR spectra investigations:**

The FTIR spectra of βCD and all stages of synthesis are presented in Figure S1 and enlarged for better visualization of details in Figure S2.

The details of FTIR spectrum of *PMA-OH* was presented in our previously published paper [1]. Briefly, the related peaks in Figure S1, are located at: 1100-1500 cm^-1^ (stretching of **C–O–C** groups of anhydride rings and **C–O** group of mercaptoethanol); 1548-1992 cm^-1^ (symmetric and asymmetric stretching band of **C=O** groups of anhydrides); 2700-3000 cm^-1^ and 3000-3605 cm^-1^ (stretching of **C–H** in **-CH**, -**CH_2_** groups and –**OH** groups of rings and mercaptoethanol, respectively). According to *PMA-OH* spectrum in Figure S1, vanishing of alkene peaks despite remaining of anhydride peaks, shows the maleic anhydride molecules were polymerized from their double band without any ring opening.

The main peaks of pure βCD in Figure S1, are appeared at: 707 and 767 cm^−1^ (βCD's skeleton); 991-161 cm^−1^ (**C–O** stretching of **C–OH** group and **C–O–C** ether band between ring units of βCD); 1647 cm^−1^ (**H–O–H** deformation band because of existing of water in βCD); 2931 cm^−1^ (**C**–**H** asymmetric-symmetric stretching of -**CH_2_** and **-CH** groups) and 3415 cm^−1^ broad peak (stretching of –**OH** group).

Unfortunately, the main peaks of βCD in spectrum of the second stage of synthesis (β*CD-g-PMA-OH*), Figure S1, overlapped with ester and carboxylic acid peaks that weakens proof of presence of βCD. The two unique peaks that appeared at 694 and 765 cm^−1^ correspond to the βCD's skeleton peaks. The peaks of hydroxy terminated poly maleate are present at: 1012-1097 cm^−1^ and 1363-1413 cm^−1^ (stretching of **C–O** group of esters and carboxylic acids, respectively); 1564-1708 cm^−1^ (stretching of **C=O** of esters and carboxylic acids); 2931 and 3408 cm^-1^ (stretching of **CH**, **CH_2_** groups and **-OH** groups, respectively). Existence of carboxylic acids and the βCD peaks associated with vanishing of anhydride signals showed that the synthesis of β*CD-g-PMA-OH* is completed.

In the FTIR spectrum of β*CD-g-PMA-co-PLGA* in Figure S1, the peaks at 1024-1454 cm^−1^ were related to stretching of **C–O** group of βCD and **C–O** band of esters and carboxylic acids. Peaks for stretching of **C=O** groups of esters and carboxylic acids appeared at 1760 cm^-1^. The peaks related to **C**–**H** stretching in **CH**, **CH_2_**, and **CH_3_** groups, were observed at 2943-3003 cm^-1^. The broad peak at 3415 cm^-1^, corresponded to **-OH** band of βCD, carboxylic acids and PLGA end **-OH** group.


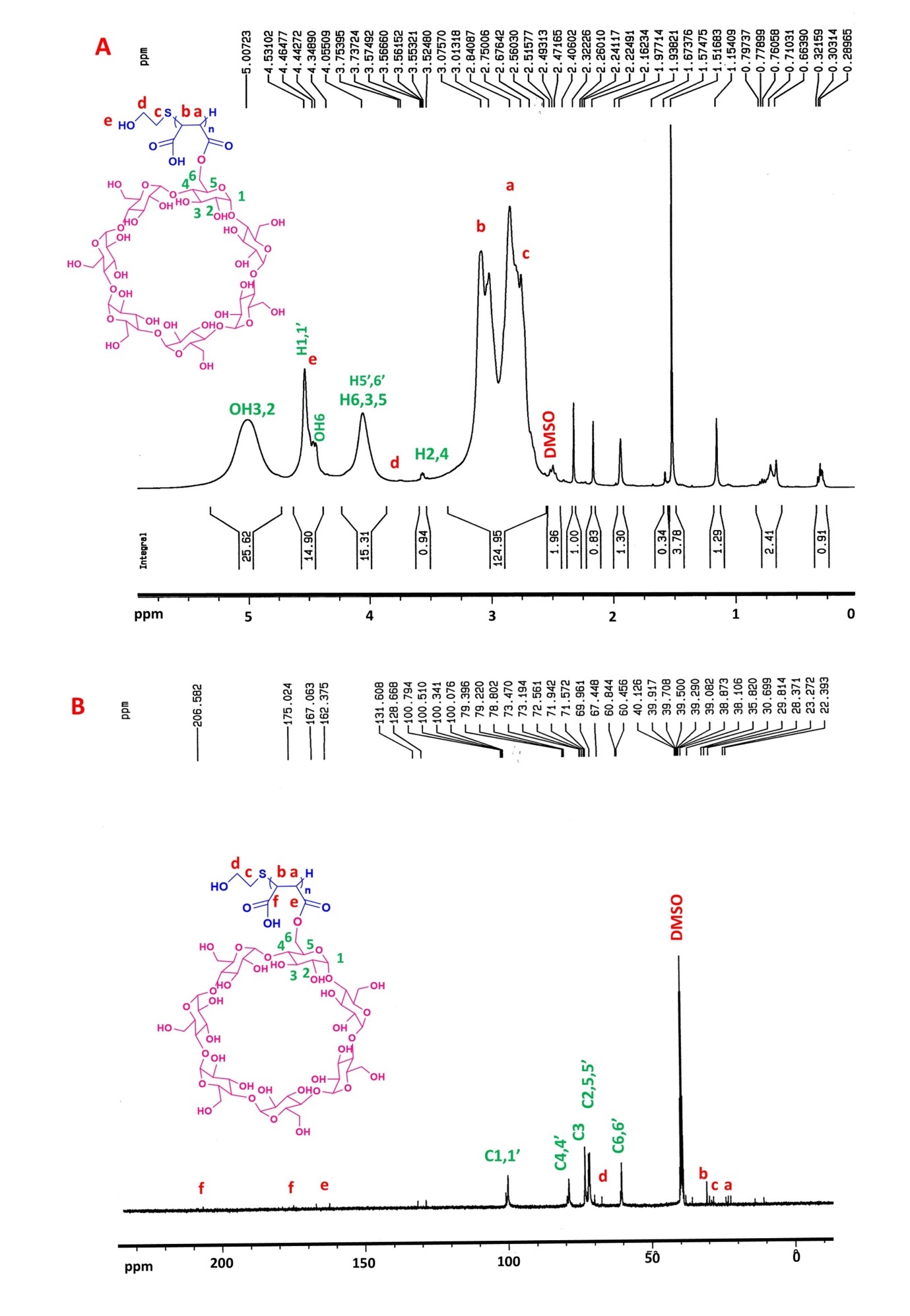


Figure S3: A) ^1^HNMR, B) ^13^CNMR spectra of βCD-g-PMA-OH

**Detailed explanation of NMR results:**

Results and detailed discussion of ^1^HNMR and ^13^CNMR spectra of PMA-OH, were presented in our previously published paper in detail [1]. The ^1^HNMR and ^13^CNMR spectra of β*CD-g-PMA-OH*, are shown in Figure S3-A and S3-B, respectively. According to ^1^HNMR spectrum of β*CD-g-PMA-OH*, the poly maleate signs are presented at: δ = 2.84 ppm (-C**H**(C(=O)-O-βCD)-CH(COOH)-); δ = 3.01, 3.07 ppm (-CH(C(=O)-O-βCD)-C**H**(COOH)-). Furthermore, the βCD peaks are observed at: δ = 3.55, 3.56 ppm (H4); 3.56, 3.57 ppm (H2); 4.05 ppm (H3, H5, H6); 4.44-4.53 ppm (OH-6, H1); 5.0 ppm (OH-2,3). The signs of mercapto end-group appeared at: δ = 2.67, 2,75 ppm (-S-C**H_2_**-CH_2_-OH); δ = 3.73, 3.75 ppm (-S-CH_2_-C**H_2_**-OH); δ = 4.34 ppm (-S-CH_2_-CH_2_-O**H**).

The ^13^CNMR spectrum of β*CD-g-PMA-OH*, revealed that the peaks of poly maleate block are located at: δ = 22.39-24.0 ppm (-**C**H(C(=O)-O-βCD)-CH(COOH)-); δ = 30.69-38.1 ppm (-CH(C(=O)-O-βCD)-**C**H(COOH)-); δ = 162.37, 167.06 ppm (-CH(**C**(=O)-O-βCD)-CH(COOH)-); δ = 175.02, 206.58 ppm (-CH(C(=O)-O-βCD)-CH(**C**OOH)-). In addition, the peaks of βCD, are obtained at: δ = 60.45, 60.84 ppm (C6,6'); 69.96, 71.57 ppm (C5, 5'), 71.94, 72.56 ppm (C2,2'); 73.19, 73.47 ppm (C3,3'); 78.80-79.39 ppm (C4,4'); 100.07-100.79 ppm (C1,1'). Moreover, the mercapto group signs appeared at: δ = 28.37-29.81 ppm, (PMA-S-**C**H_2_-CH_2_-OH); δ = 67.44 ppm, (PMA-S-CH_2_-**C**H_2_-OH).

The ^1^HNMR and ^13^CNMR spectra of β*CD-g-PMA-co-PLGA*, are presented in Figure 3-A and 3-B, respectively. Based on the ^1^HNMR spectrum in Figure 3-A, the peaks related to lactide section of PLGA block were observed at: δ=1.23-1.38 ppm (end C**H**_3_ group); δ=1.47 ppm (CH_3_); δ=5.11-5.26 ppm (C**H**). Similarly, the signs of glycolide were located at: δ=4.72-ppm (end -O**H** group); 4.86-4.91 ppm (C**H**_2_). The maleate block signs were seen at: δ=2.85-2.95 ppm (-C**H**(C(=O)-O-βCD)-CH(COOH)-); 3.36 ppm (-CH(C(=O)-O-βCD)-C**H**(COOH)-); 4.21 ppm (-S-(C**H**(COOH))-CH(C(=O)-O-βCD)-); 12.776 ppm (a broad peak between 11-14 ppm, -CH(C(=O)-O-βCD)-CH(COO**H**)-). The signatures of βCD were observed at: δ = 3.47 ppm (H4, overlapped with δ=3.36 ppm peak of maleate), 3.51-3.59 ppm (H2, H5), 3.65 ppm (H3), 3.76 ppm (H6); 3.91 ppm (H5'), 4.11-4.15 ppm (H6'); 4.58 ppm (OH6); 4.76-4.84 ppm (H1,1'); 5.40-5.50 ppm (OH2,3). The signs of mercapto unit were observed at: δ= 2.71-2.73 ppm (βCD-g-PMA-S-C**H_2_**-CH_2_-O-PLGA); 4.62, 4.64 ppm (βCD-g-PMA-S-CH_2_-C**H_2_**-O-PLGA) that overlapped with βCD peaks. According to ^13^CNMR spectrum of β*CD-g-PMA-co-PLGA*, the signs of lactide units of PLGA were seen at: δ=16.42-16.49 ppm (**C**H_3_); δ=68.67-68.88 ppm, (**C**H); δ=168.97-169.68 ppm, (-CH(CH_3_)-**C**(=O)-O-CH(CH_3_)-). Moreover, the peaks related to glycolide were detected at: δ=60.44, 60.7 ppm (**C**H_2_); δ=166.56-167.21 ppm (-CH_2_-**C**(=O)-O-CH_2_-). The signatures of poly maleate block were observed at: δ=20.31-23.25 ppm (-**C**H(-C(=O)-O-βCD)-CH(COOH)-); 40.41 ppm (-CH(-(=O)-O-βCD) -**C**H(COOH)-); δ=172.02 ppm, (-CH(-**C**(=O)-O-βCD)-CH(COOH)-); δ=174.0 ppm (-CH(C(=O)-O-βCD)-CH(-**C**(=O)-OH)-). βCD peaks were observed at: δ = 59.27-59.97 ppm (C6,6'); 71.55 ppm (C5), 71.992 ppm C2); 2.53, 73.18 ppm (C3,3'); 78.75-79.45 ppm (C4,4'); 100.02-100.77 ppm (C1,1'). The peaks related to mercapto were detected at: δ=28.35-29.79 ppm, (βCD-g-PMA-S-**C**H_2_-CH_2_-O-PLGA); and 67.86-68.03 ppm (βCD-g-PMA-S- CH_2_-**C**H_2_-O-PLGA).

**Calculation of copolymer molar mass using data of ^1^HNMR spectra**

The ^1^HNMR spectra was used for estimation of molar mass (M_n_) of the copolymer. Therefore, the results of integration of the peaks related to each monomer were used according to following equations [2].

$\boldsymbol{n}_{\boldsymbol{polymer}}\boldsymbol{=}\frac{\sum_{\boldsymbol{i=1}}^{\boldsymbol{m}} \frac{\boldsymbol{I}_{\boldsymbol{i}}}{\boldsymbol{p}_{\boldsymbol{i}}}}{\boldsymbol{m}}$ **Equation (4)**

$\boldsymbol{M}_{\boldsymbol{n}}\boldsymbol{=n. (monomers molecular mass)}$ **Equation (5)**

In the equation 4, "*m*" is the number of used peaks of copolymer, and "*p_i_*" and "*I_i_*" are the number and integration of protons that related to *i^th^* signal of copolymer.

The integration value of H5' peak of βCD at δ = 3.91 ppm (equal to ≈ 0.07), is selected equal to one hydrogen integration. Then, the other peaks integration amount was divided by H5' integration value.

For example: Lactide -CH_3_ peak integration value at δ = 1.47 ppm, 6H for two -CH_3_ group per molecule, was 6.43 that is divided by 0.07 (H5' integration value). Then, the result (91.86 ≈ 92), was used for determining mole of lactide monomer in copolymer, according to above formula:

$$n_{Lactide}=\frac{\frac{92}{6}}{1}=15.3\approx15$$

**GL**: δ = 4.86-4.91 ppm (CH_2_), 4H, A≈ 2.02; thus: 2/(0.07) ≈ 29 (if the peak area related to H1 of βCD is ignored):

$$n_{Glycolide}=\frac{\frac{29}{4}}{1}\approx7$$

**MA**: δ = 3.36 ppm (-CH(-C(=O)-O-βCD)-CH(COOH)-), 1H, **βCD**: δ = 3.47 ppm (H4 of **βCD**), 7H for **βCD** ring, at all this peak area was related to 8 hydrogen (1H of **MA** and 7H of **βCD**). A≈ 2.4; thus: 2.4/ (0.07) = 34:

(Hypothesis: 3 out of 4 moles are related to MA and one mole related to **βCD**, according to **β**CD following calculation). Therefore: 4-1=3 moles for MA are considered.

$$n_{MA}=\frac{\frac{34}{8}}{1}=4$$

**βCD**: δ = 5.40-5.50 ppm (OH-2,3), 2H and 14 H for βCD ring, A≈ 0.74; thus: 0.74/ (0.07) = 11:

$$n_{\beta CD}=\frac{\frac{11}{14}}{1}=0.8\approx1$$

**ME**: δ = 2.71-2.73 ppm (βCD-g-PMA-S-C**H_2_**-CH_2_-O-PLGA), 2H, A≈ 0.03; thus: 0.03/ (0.07) = 0.4:

$$n_{ME}=\frac{\frac{0.4}{2}}{1}=0.2$$

Finally, considering above calculated moles of copolymer sections, the copolymer molar mass is calculated according to Equation 5:

$$M_{n}=\left( 15\times144.13 \right)+\left( 7\times116.07 \right)+\left( 3\times98.06 \right)+\left( 1\times1134.98 \right)+\left( 0.2\times78.13 \right)+1 =4420.23 g/mol$$

The last number (1) is related to end -H group of copolymer.


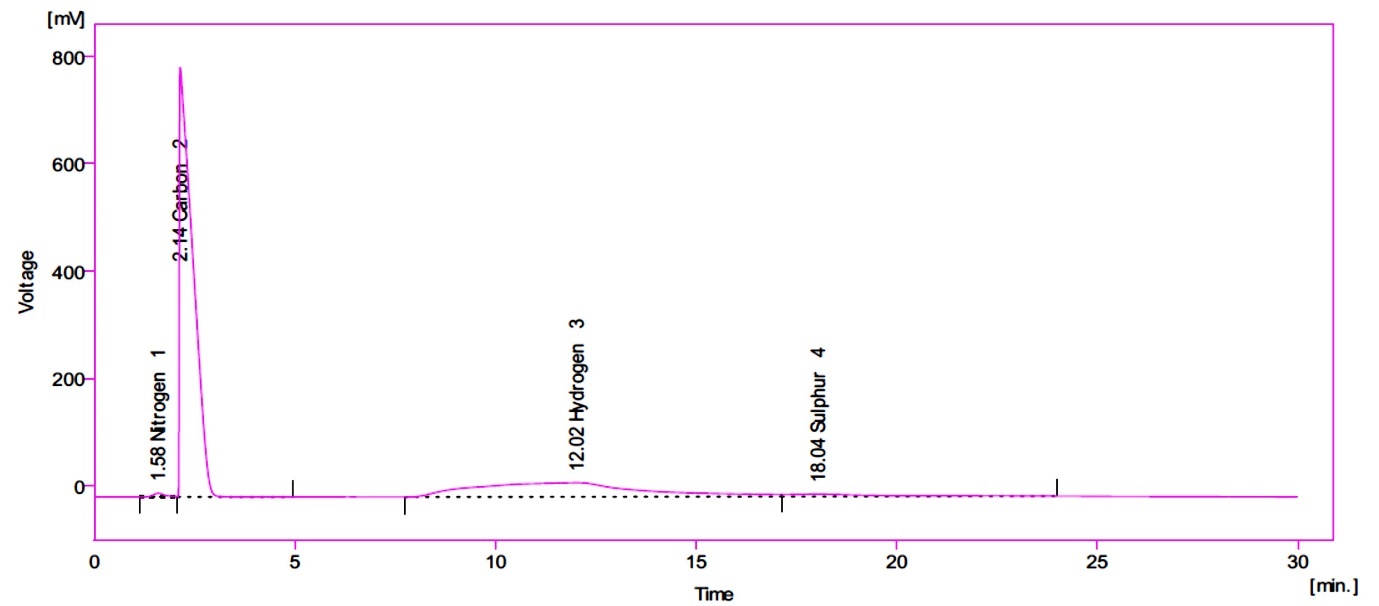


Figure S4: Result of CHNS elemental analysis as time (min) versus voltage (mV) plot

Table S2: Results of CHNS-O elemental analysis of βCD-g-PMA-co-PLGA copolymer.

| CHNS | Elemental analysis (W %) | | | | |
| --- | --- | --- | --- | --- | --- |
|  | **C** | **H** | **O** | **S** | **N** |
| Copolymer | 52.55 | 6.83 | 35.32 | 4.44 | 0.85 |


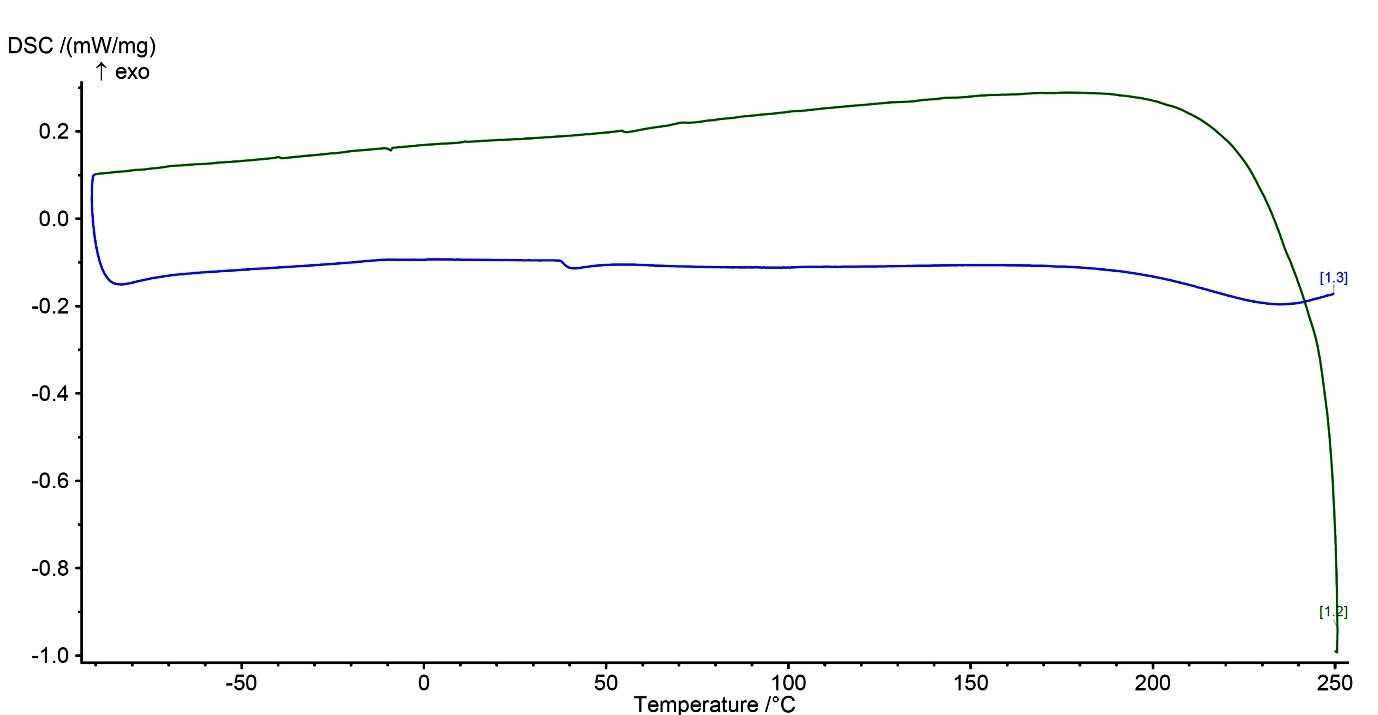


Figure S5: Results of DSC test of βCD-g-PMA-co-PLGA copolymer as temperature against heat flow plot


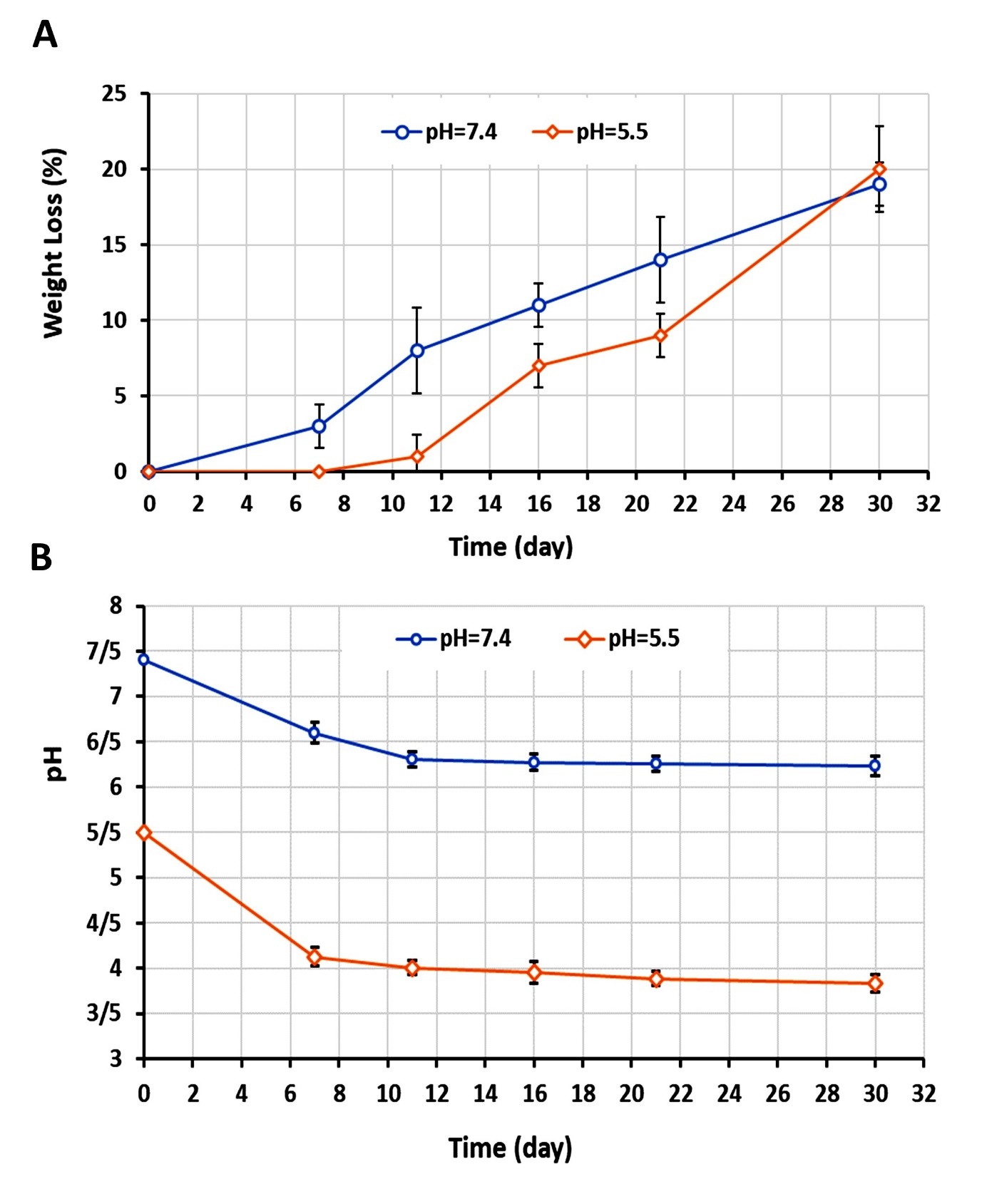


Figure S6: Results of βCD-g-PMA-co-PLGA copolymer in-vitro degradation test in PBS. A) Diagram of weight loss (WL %) of copolymer versus time (day) with initial pH of 7.4 and 5.5, B) Plot of pH variation (PBS as media containing copolymer with initial pH = 7.4 and 5.5) versus time (day).


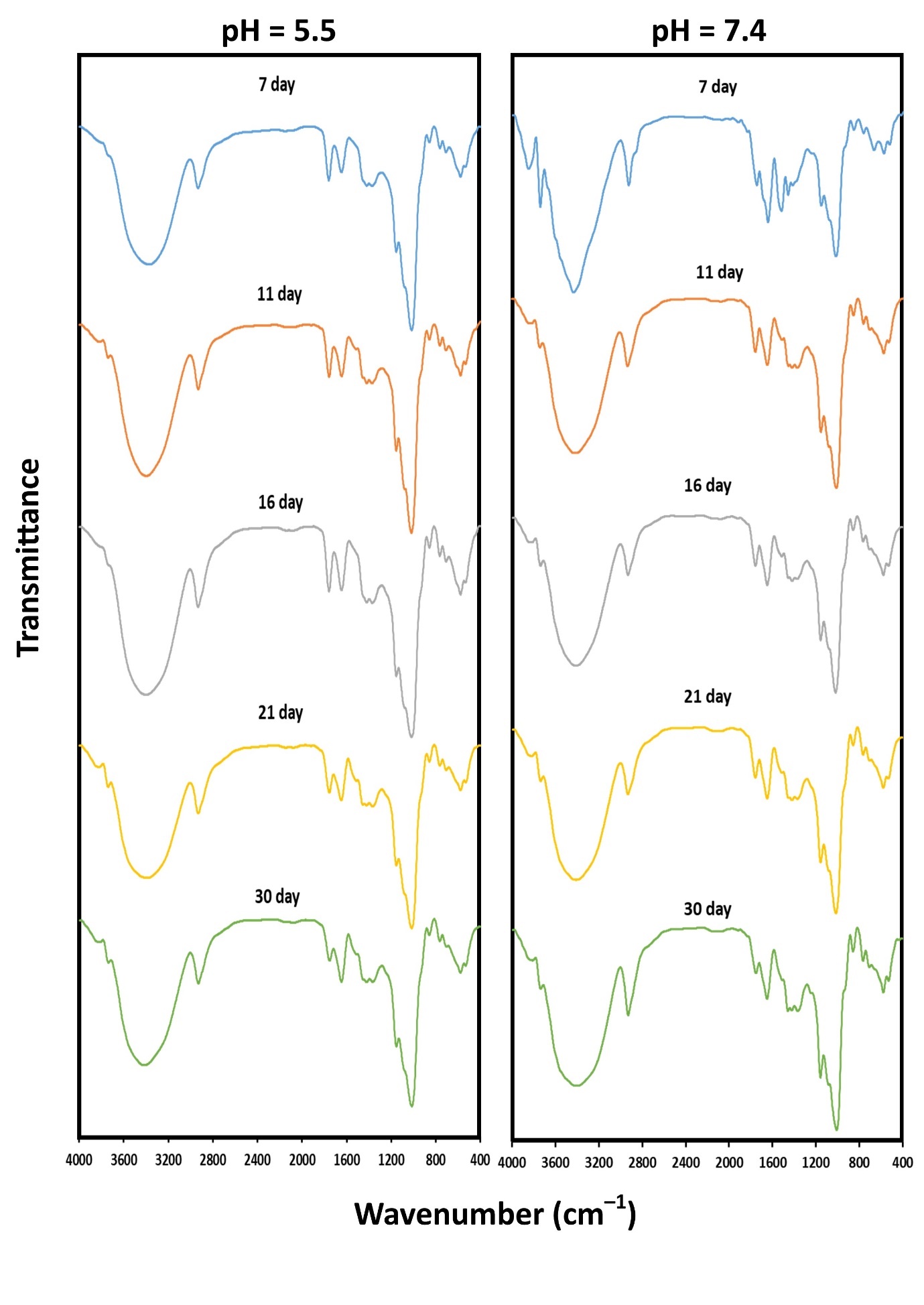


Figure S7: FTIR Spectra of βCD-g-PMA-co-PLGA copolymer in degradation test in PBS at various time intervals (7, 11, 16, 21, 30 days) with initial pH of 7.4 and 5.5.


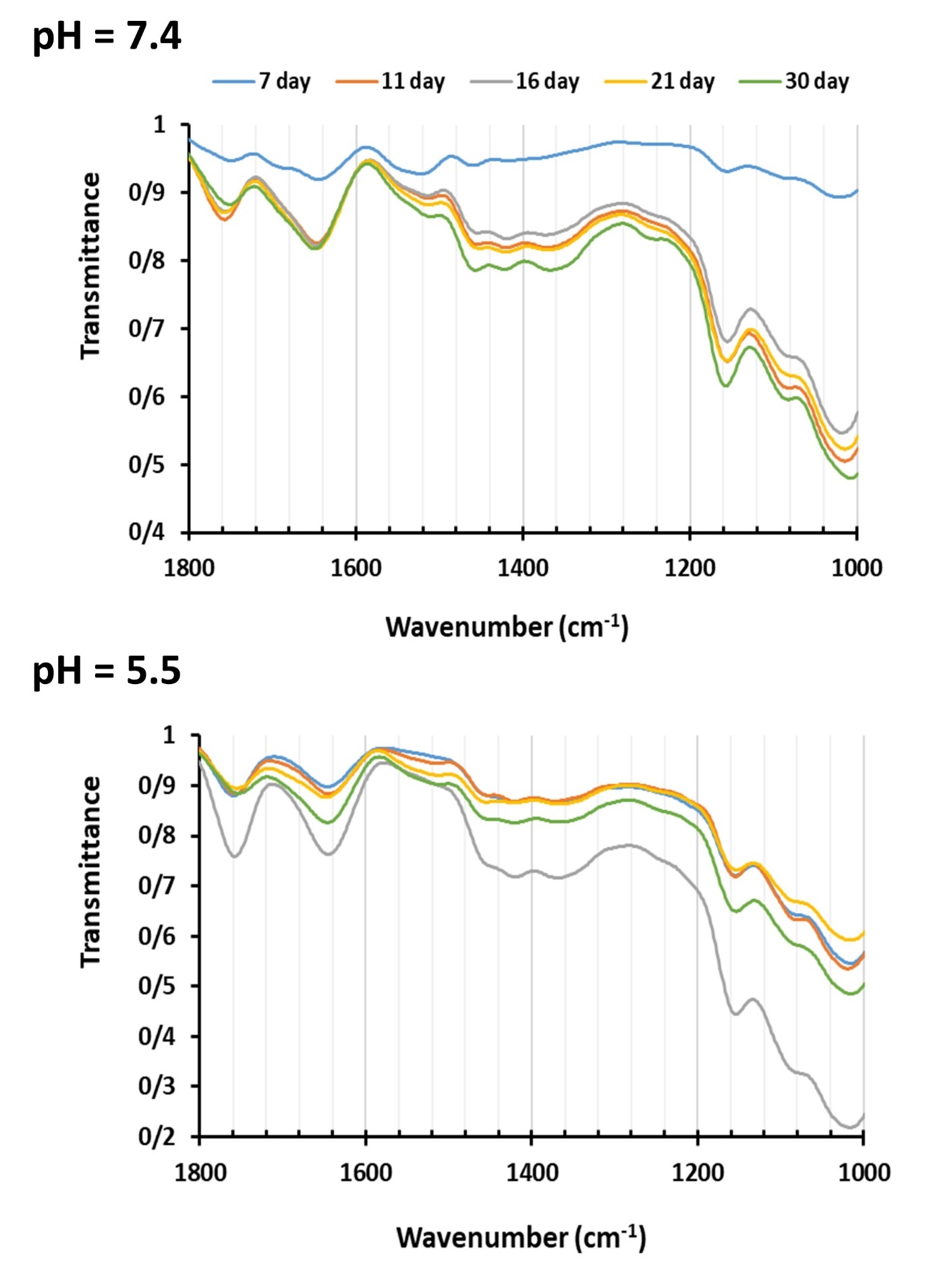


Figure S8: Enlarged FTIR Spectra (1000-1800 cm^-1^) of βCD-g-PMA-co-PLGA copolymer in degradation test in PBS media at various time intervals (7, 11, 16, 21, 30 days) with initial pH of 7.4 and 5.5

**Detailed explanation of FTIR spectra of copolymer degradation test:**

According to Figures S7 and S8, no noticeable variation could be seen in the main peaks. However, some little peaks appeared at 1515-1552 cm^-1^. Furthermore, the intensity of peak at 1760 cm^-1^ (**C=O**) is reduced and shifted to 1749 cm^-1^, whereas intensity of 1645 cm^-1^ peak is increased. These new peaks and variation in carboxylic acid and ester peaks probably are because of degradation products such as aldehyde, ketones and hydrolyzed carboxylic acids groups. The peaks of **C=O** stretching band of aldehyde and ketones appeared at (1700-1750 cm^-1^), that have lower frequencies in comparison with **C=O** band of ester groups (1755-1765 cm^-1^)


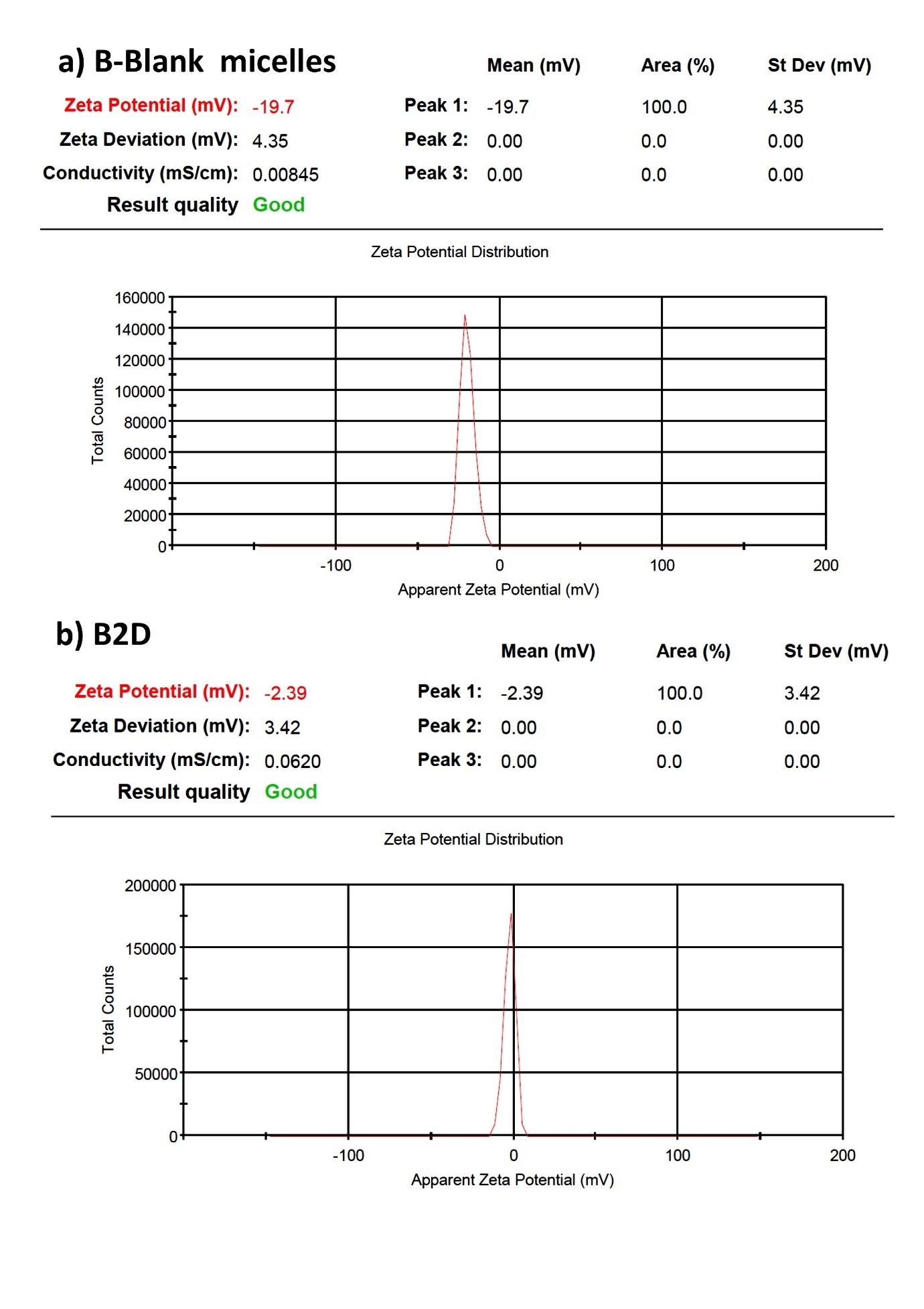


Figure S9: Zeta-potential of a) **B**-blank βCD-g-PMA-co-PLGA micelles (**PB)** and b) co-drug loaded βCD-g-PMA-co-PLGA micelles (**B2D)**, by DLS-Zeta test.


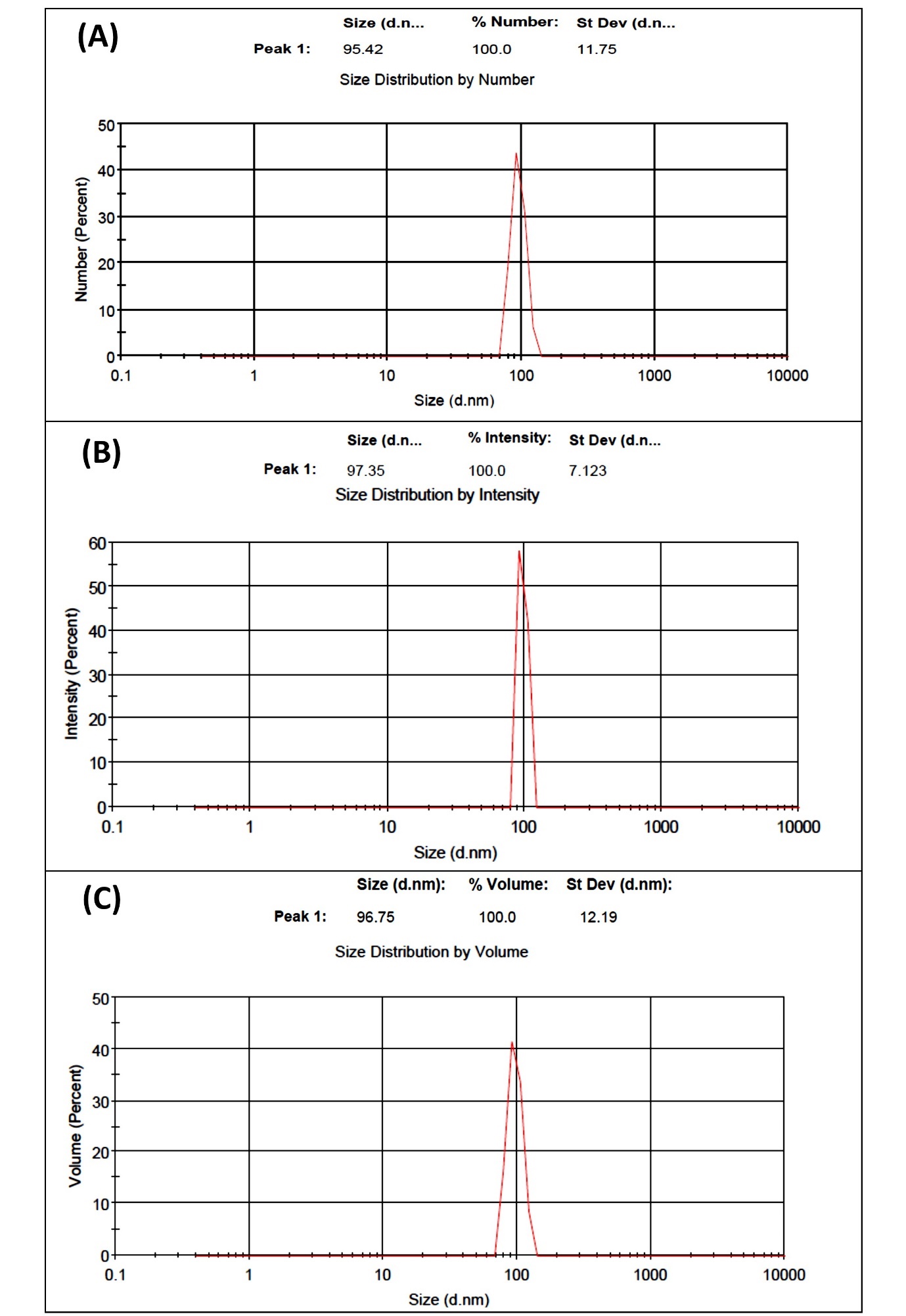


Figure S10: DLS results of the blank βCD-g-PMA-co-PLGA micelles (**PB**) A) by number, B) by intensity and C) by volume versus Size (d.nm)


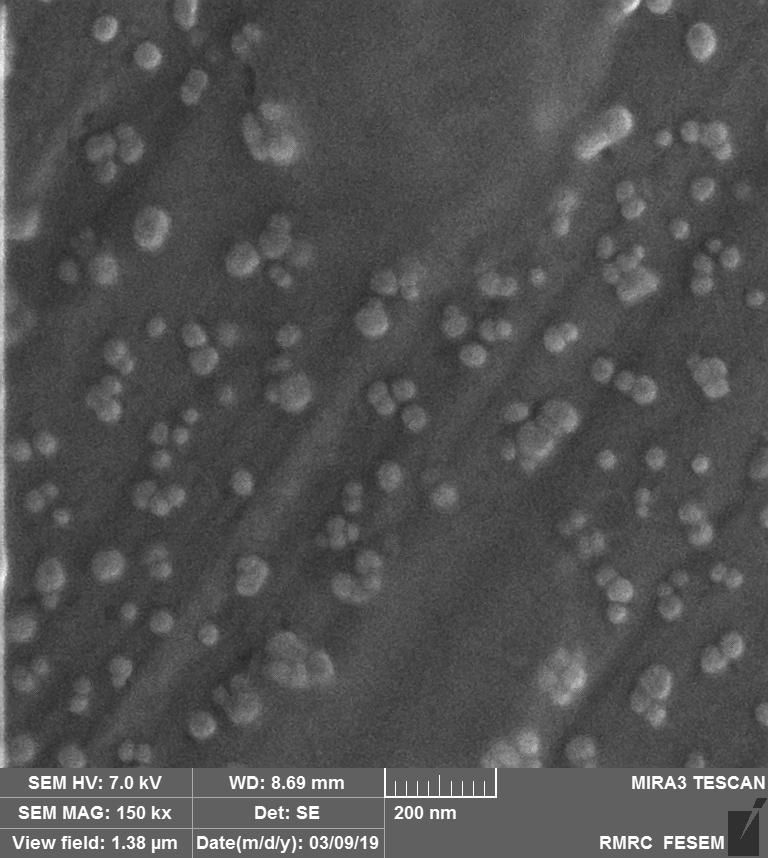


Figure S11: SEM image of blank βCD-g-PMA-co-PLGA micelles (**PB**) with concentration of about 25 μg/mL.

Table S3: IC_50_ dosage of formulations calculatied by GraphPad prism software using MTT results (n=3, P<0.05)

| *Formulations* | B2D | 2D | BD | Dox | BC | Conf |
| --- | --- | --- | --- | --- | --- | --- |
| *IC_50_ (μg/mL)* | 0.259  0.1295 μg/mL of Dox  0.1295 μg/mL of Conf | 1.594  0.797 μg/mL of Dox  0.797 μg/mL of Conf | 0.408 | 0.377 | 3.567 | 30.887 |


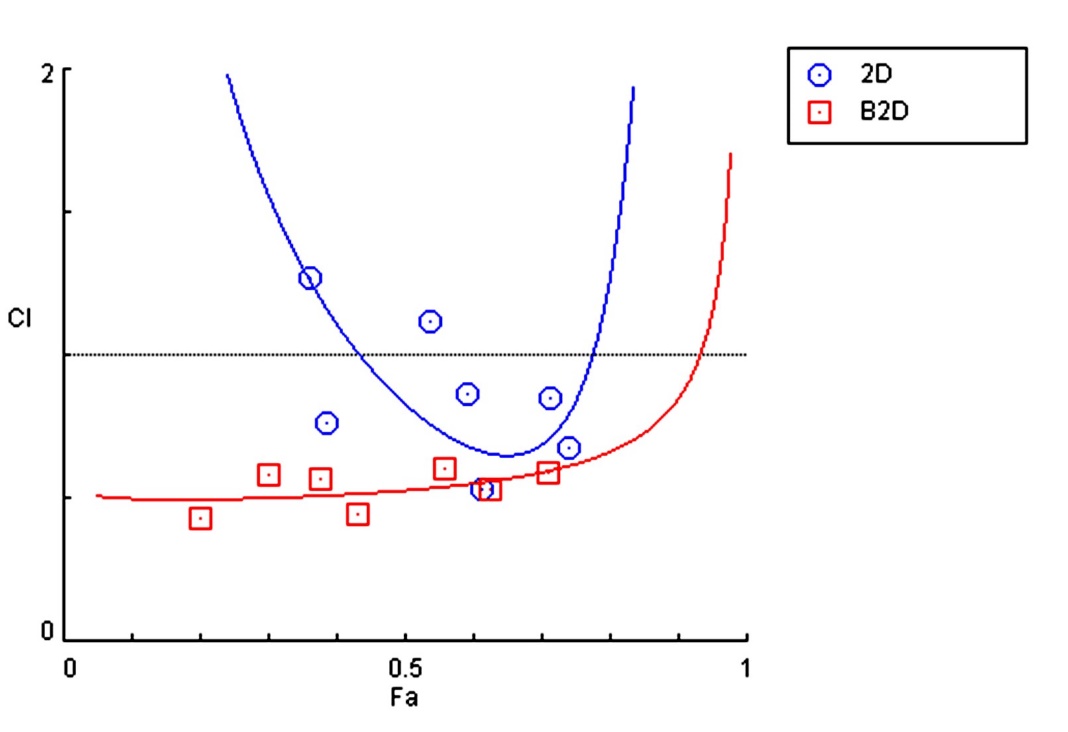


Figure S12: Combination index plot of **B2D** and **2D**, prepred by CompuSyn software. (Abbreviations: Co-drug loaded βCD-g-PMA-co-PLGA micelles: **B2D**; Free Doxorubicin-Conferone:**2D**).

Table S4: Results of combination index (CI) of **B2D** and **2D** with different doses (calculated by CompuSyn software). (Abbreviations: Co-drug loaded βCD-g-PMA-co-PLGA micelles: **B2D**; Free Doxorubicin-Conferone:**2D**).

| Total dose (μg/mL) | B2D | | 2D | |
| --- | --- | --- | --- | --- |
|  | **Fa** | **CI** | **Fa** | **CI** |
| 0.078 | 0.711 | 0.588 | 0.741 | 0.6766 |
| 0.156 | 0.626 | 0.532 | 0.713 | 0.8508 |
| 0.312 | 0.559 | 0.607 | 0.614 | 0.5294 |
| 0.625 | 0.433 | 0.455 | 0.591 | 0.8633 |
| 1.25 | 0.377 | 0.565 | 0.537 | 1.1186 |
| 2.5 | 0.301 | 0.585 | 0.387 | 0.7661 |
| 5 | 0.203 | 0.428 | 0.361 | 1.2704 |

| formulations | Cell cycle (%) | | | |
| --- | --- | --- | --- | --- |
|  | **Sub G_1_** | **G_0_/G_1_** | **S** | **G_2_/M** |
| B2D | 1.15 | 3.65 | 8.82 | 86.40 |
| 2D | 0.64 | 1.07 | 3.22 | 95.00 |
| BD | 1.73 | 11.40 | 19.50 | 67.00 |
| Dox | 1.17 | 2.25 | 9.44 | 87.00 |
| BC | 0.33 | 26.40 | 51.30 | 22.00 |
| Conf | 0.98 | 61.00 | 15.80 | 22.20 |
| PB | 1.21 | 70.80 | 7.06 | 21.00 |
| Control | 0.44 | 73.50 | 10.80 | 15.30 |

Table S5: Results of cell cycle analysis of MDA-MB-231 cells in presence of all the nano-formulations (**PB**, **B2D**, **BD** and **BC** ) and formulations (**2D**, **Dox** and **Conf**) by flow cytometry; untreated cells were used as the control group. (Abbreviations: Blank βCD-g-PMA-co-PLGA micelles: **PB**; Co-drug loaded βCD-g-PMA-co-PLGA micelles: **B2D**; Doxorubicin loaded βCD-g-PMA-co-PLGA micelles: **BD**; Conferone loaded βCD-g-PMA-co-PLGA micelles: **BC**; Free Doxorubicin-Conferone:**2D**; Free Doxorubicin:**Dox**; Free Conferone:**Conf**).

Table S6: The MDA-MB-231 cells apoptosis percentage in the presence of the nano-formulation (**PB**, **B2D**, **BD** and **BC**) using flow cytometry. (Abbreviations: Blank βCD-g-PMA-co-PLGA micelles: **PB**; Co-drug loaded βCD-g-PMA-co-PLGA micelles: **B2D**; Doxorubicin loaded βCD-g-PMA-co-PLGA micelles: **BD**; Conferone loaded βCD-g-PMA-co-PLGA micelles: **BC**).

| Formulations | Q4: Viable cell % | Q3: Early apoptosis % | Q2: Late apoptosis % | Q1: Necrosis % |
| --- | --- | --- | --- | --- |
| B2D | 0.0 | 0.0 | 98.7 | 1.33 |
| BD | 3.02 | 9.3E-3 | 83.0 | 14.0 |
| BC | 82.2 | 3.43 | 10.8 | 3.66 |
| PB | 83.4 | 5.81 | 8.56 | 2.22 |
| Control | 94.3 | 4.14 | 1.47 | 0.062 |

Table S7: Results of western blot test of MDA-MB-231 cells treated by co-drug loaded βCD-g-PMA-co-PLGA micelles (**B2D**) and fold changes of Bcl-2, Bax, pro-Caspase9, cleaved-Caspase9, pro-Caspase3, cleaved-Caspase3, pro-Caspase7, cleaved-Caspase7 and p27 and p53 proteins compared to the control group (protein expression = 1)

| Fold changes | proteins | | | | | | | | | |
| --- | --- | --- | --- | --- | --- | --- | --- | --- | --- | --- |
|  | **Bcl-2** | **Bax** | **Pro-Casp9** | **Cl-Casp9** | **Pro-Casp3** | **Cl-Casp3** | **Pro-Casp7** | **Cl-Casp7** | **p27** | **p53** |
| B2D | 0.673 | 1.75 | 0.369 | 5.413 | 0.563 | 14.00 | 0.342 | 22.547 | 3.202 | 2.867 |

**References:**

[1] A. Rahmani, H. Zavvar Mousavi, R. Salehi, A. Bagheri, Novel pH-sensitive and biodegradable micelles for the combined delivery of doxorubicin and conferone to induce apoptosis in MDA-MB-231 breast cancer cell line, RSC Adv. 10 (2020) 29228–29246. https://doi.org/10.1039/D0RA03467C.

[2] J.W. Wackerly, J.F. Dunne, Synthesis of Polystyrene and Molecular Weight Determination by 1H NMR End-Group Analysis, J. Chem. Educ. 94 (2017) 1790–1793. https://doi.org/10.1021/acs.jchemed.6b00814.
